# Numerical Investigation of a Pt/HfSiON/Ti MIM Rectifying Diode for LWIR Energy Harvesting

**DOI:** 10.3390/nano16181159

**Published:** 2026-09-15

**Authors:** Rocco Citroni, Luca Balestreri, Fabio Mangini, Fabrizio Frezza

**Affiliations:** 1 Department of Information Engineering, Electronics and Telecommunications (DIET), “La Sapienza” University of Rome, 00184 Rome, Italy; luca.balestreri@uniroma1.it (L.B.); fabrizio.frezza@uniroma1.it (F.F.); 2 Department of Engineering, Niccolò Cusano University, 00166 Rome, Italy; fabio.mangini@unicusano.it

**Keywords:** HfSiON, metal–insulator–metal (MIM) diode, long-wave infrared (LWIR), infrared energy harvesting, Simmons tunneling model, antenna–figure of merit (FOM)

## Abstract

This work presents a numerical investigation of an asymmetric Pt/HfSiON/Ti metal–insulator–metal (MIM) tunnel diode for long-wave infrared (LWIR) rectenna applications at 28.3 THz (10.6 μm). HfSiON is investigated as the tunneling dielectric owing to its favorable electronic properties, thermal stability, and compatibility with nanoscale device fabrication. The electrical transport and rectification characteristics are evaluated using the full Simmons quantum-mechanical tunneling model implemented in MATLAB release 2025b. The analysis encompasses the current density–voltage (J–V) and current–voltage (I–V) characteristics, zero-bias dynamic resistance, current asymmetry, nonlinearity, responsivity, and temperature dependence. Under AC excitation, the Pt/HfSiON/Ti diode exhibits a calculated rectified current density of 1.78 × 10^2^ A/cm^2^ at zero DC bias, while a current density of 6.32 × 10^5^ A/cm^2^ is obtained at an applied voltage amplitude of ±0.5 V. The asymmetric electrode configuration, arising from the difference in the work functions of Pt and Ti, results in a calculated asymmetry of 2.5 × 10^4^. At zero DC bias, the diode exhibits a zero-bias dynamic resistance of 3.85 × 10^5^ Ω and a zero-bias responsivity of approximately 10 V^−1^. The calculated rectification characteristics show only weak sensitivity to temperature over the investigated range, indicating that the transport response is predominantly governed by quantum-mechanical tunneling rather than thermally activated processes. These results demonstrate the potential of HfSiON as a tunneling dielectric for nanoscale MIM rectifiers and indicate that the asymmetric Pt/HfSiON/Ti architecture provides strong nonlinear rectification and favorable zero-bias response for LWIR rectenna and energy-harvesting applications.

## 1. Introduction

The development of self-powered sensors and distributed sensing platforms has increased the demand for energy-harvesting technologies capable of converting ambient electromagnetic radiation into usable electrical power. In particular, self-powered sensing nodes and autonomous electronic devices require reliable and continuously available energy sources to operate without external power supplies or frequent battery replacement. In this context, harvesting energy directly from the surrounding electromagnetic environment represents a promising approach for enabling maintenance-free operation, extending device lifetime, and facilitating the deployment of large-scale distributed sensor networks. While solar energy harvesting remains one of the most established approaches, its effectiveness is strongly dependent on illumination conditions and is significantly reduced or eliminated during nighttime, in shaded environments, or in enclosed and indoor settings. In contrast, long-wave infrared (LWIR) radiation is continuously emitted by objects and surfaces as a consequence of their thermal energy and is therefore available under a broader range of environmental conditions, even in the absence of direct sunlight. LWIR harvesting is consequently attractive for autonomous and self-powered electronic systems requiring operation beyond daylight-dependent conditions. Moreover, the LWIR spectral range is particularly relevant for thermal radiation from the surroundings and for applications involving waste-heat recovery, enabling the possibility of converting otherwise unused thermal radiation into electrical power. These characteristics motivate the investigation of LWIR rectennas as a complementary approach to conventional solar-based energy harvesting for next-generation autonomous sensing and low-power electronic systems. The infrared-frequency rectifying antenna, or rectenna, is based on electromagnetic coupling followed by electrical rectification. The receiving antenna is designed to efficiently absorb electromagnetic radiation at a central wavelength of 10.6 μm, corresponding to a frequency of approximately 28.3 THz. A wavelength of 10.6 µm represents the peak of thermal infrared radiation emitted by both the Earth and any object on its surface, consistent with Wien’s displacement law for ambient terrestrial temperatures.

This wavelength is particularly relevant for both experimental and theoretical investigations of rectenna devices [1,2,3]. The rectification process represents the main bottleneck and an active area of research in rectenna technology. While building efficient antennas is a mature field, converting the high-frequency alternating current (AC) captured by the antenna into useful direct current (DC) electricity introduces severe physical limitations. At infrared and optical frequencies, conventional semiconductor p–n or Schottky diodes become increasingly unsuitable because their carrier transit and junction response cannot readily follow the extremely high-frequency excitation. MIM tunnel diodes provide an alternative mechanism based on quantum-mechanical electron tunneling through an ultrathin dielectric barrier. Since tunneling occurs on an ultrafast timescale, MIM structures are theoretically capable of responding to frequencies far beyond the operating range of conventional rectifying junctions. Their nanoscale dimensions, compatibility with planar fabrication, and possibility of zero-bias operation make them particularly attractive for optical and infrared rectennas [2,3,4]. The performance of an MIM rectifier is, however, determined by a strong interplay between tunneling transport, material properties, and high-frequency impedance matching.

Several figures of merit (FOM) are commonly used to assess the suitability of an MIM diode for rectenna applications, including the zero-bias dynamic resistance R_0_, responsivity S_0_, nonlinearity, asymmetry, and the RC-limited cutoff frequency. High responsivity and strong nonlinearity are required to obtain efficient AC-to-DC conversion, whereas sufficiently low resistance and capacitance are necessary to enable effective coupling to the receiving antenna [3,4,5,6].

Consequently, optimizing an MIM diode is inherently a multidimensional problem. Reducing the dielectric thickness can significantly increase the tunneling current and lower the resistance, but excessive tunneling may compromise rectification. Conversely, increasing the dielectric thickness can suppress the current and increase the resistance. Similarly, the dielectric permittivity influences the junction capacitance and therefore the high-frequency response. Recent optimization studies have explicitly demonstrated the importance of simultaneously considering barrier heights, dielectric constants, metal work functions, resistance, and responsivity rather than optimizing a single diode parameter in isolation [5,6]. The choice of the tunneling dielectric is therefore a key design parameter in MIM rectifiers [3,7]. Al_2_O_3_ has been extensively investigated because of its established fabrication technology, relatively low dielectric permittivity, and suitability for nanoscale tunnel barriers. A pioneering experimental demonstration of a passive rectenna at 28.3 THz employed an Au/Al_2_O_3_/Ti diode integrated into a bow-tie antenna. The use of different work-function metals enabled zero-bias operation, while the low-permittivity Al_2_O_3_ barrier helped reduce the junction capacitance and associated RC limitation. The experiment demonstrated optical rectification at 10.6 μm using a CO_2_ laser, establishing 28.3 THz as a realistic operating frequency for MIM-based infrared rectennas rather than merely a theoretical target [1]. Despite this important demonstration, the performance of single-insulator MIM structures remains constrained by the trade-off between low resistance, high nonlinearity, and low capacitance. In particular, although Al_2_O_3_ can provide a low-capacitance junction, its relatively large tunneling barrier can result in high zero-bias resistance. This resistance can substantially affect the coupling between the antenna and diode and consequently the overall rectenna efficiency [1,5,6]. Experimental and theoretical studies have therefore investigated alternative dielectric materials and multilayer barriers to modify the tunneling profile and improve the resistance–responsivity trade-off [7,8,9].

Among the alternative dielectric systems, NiO, TiO_2_, HfO_2_, Ta_2_O_5_, Nb_2_O_5_, ZnO, and Sc_2_O_3_ have been investigated in single- and multiple-insulator configurations [7,8]. In particular, multilayer metal–insulator–insulator–metal (MIIM or MI^2^M) architectures provide an additional degree of freedom because two different barriers can be engineered to produce an asymmetric tunneling potential. Such structures can exploit resonant or step tunneling mechanisms to enhance the nonlinear response while maintaining a sufficiently low resistance [8,9,10]. A systematic review of single, double-, and triple-insulator tunnel rectifiers has shown that NiO and Al_2_O_3_ have been among the most extensively investigated oxides, often combined with high-k materials such as HfO_2_, Ta_2_O_5_, Nb_2_O_5_, ZnO, and TiO_2_. The literature also demonstrates that sub-nanometer control of dielectric thickness and stoichiometry can strongly influence the resulting tunneling characteristics [7,8]. The development of MIIM structures has produced significant improvements in the simultaneous optimization of resistance and responsivity. For example, a Ni/NiO/Al_2_O_3_/CrAu double-insulator diode with a nanoscale active area achieved a resistance below 2 kΩ by engineering a nonstoichiometric Al_2_O_3_ layer and exploiting the modified tunneling barrier [11]. This approach demonstrated that dielectric stoichiometry and barrier engineering can be as important as the nominal dielectric material itself. Furthermore, optical measurements at 10.6 μm showed that resonant tunneling can enhance the rectification response at 28.3 THz, providing experimental evidence that multilayer tunnel barriers can improve infrared rectification beyond conventional single-insulator structures [9,11].

Another important research direction has been the optimization of MIIM diodes specifically for impedance matching with nanoantennas. Elsharabasy et al. performed a systematic material-level optimization of MIIM rectifiers for 10.6 μm operation and showed that the dielectric barrier heights, dielectric constants, metal work functions, and diode resistance must be optimized simultaneously [5]. Importantly, their optimization explicitly considered a target diode resistance of approximately 100 Ω to facilitate matching with the nanoantenna and improve the overall rectenna efficiency. This work highlights an essential point for infrared rectenna design: the optimum MIM diode is not necessarily the device with the highest intrinsic rectification performance, but rather the device whose electrical characteristics are compatible with the electromagnetic impedance of the antenna [5,6]. More recent studies have also investigated alternative dielectric materials in order to overcome the limitations associated with conventional Al_2_O_3_-based structures. For example, Sc_2_O_3_ has been investigated as an alternative oxide, with experimental results demonstrating improved zero-bias responsivity compared with a corresponding Au/Al_2_O_3_/Au structure [7]. Such investigations confirm that the dielectric material is not merely an insulating spacer but an active component controlling the tunneling barrier, resistance, asymmetry, capacitance, and ultimately the rectification performance [7,8].

Despite these advances, the literature remains strongly concentrated on a relatively limited set of dielectric materials and device architectures. In particular, Al_2_O_3_-based MIM structures and NiO/Al_2_O_3_-based MIIM structures dominate the experimental literature at 10.6 μm, while other technologically relevant high-k dielectric systems remain comparatively unexplored for LWIR rectification [7,8,9,11]. The available studies have demonstrated that high-k materials can provide useful control over barrier height and tunneling probability, but increasing dielectric permittivity also increases junction capacitance for a given geometry, creating a direct trade-off between tunneling transport and high-frequency operation. Thus, the identification of alternative dielectrics requires simultaneous consideration of barrier properties, dielectric permittivity, metal work functions, tunneling current, asymmetry, zero-bias dynamic resistance, and responsivity rather than evaluation of dielectric properties alone [5,6,7,8].

In this context, hafnium silicon oxynitride (HfSiON) represents an interesting but comparatively unexplored dielectric candidate for LWIR MIM rectification. HfSiON combines the moderate high-k characteristics associated with hafnium-based dielectrics with the ability to modify the electronic and dielectric properties through Si and N incorporation [12,13,14,15]. Its thermal stability and compatibility with advanced thin-film processing make it attractive for nanoscale electronic devices [12,13,14]. Nevertheless, its application as the tunneling barrier of an asymmetric MIM rectifier specifically designed for 28.3 THz (10.6 μm) operation has not been systematically established in the available rectenna literature. In particular, there is a lack of a unified numerical assessment linking the HfSiON barrier to the key rectenna FOMs—zero-bias dynamic resistance, asymmetry, nonlinearity, responsivity, and temperature dependence—within a single Platinum/Hafnium Silicon Oxynitride/Titanium (Pt/HfSiON/Ti) architecture. This gap is particularly relevant because the choice of electrode materials must be considered together with the dielectric barrier. The use of dissimilar metals provides an intrinsic asymmetry in the tunneling potential through their different work functions, which can promote zero-bias rectification [1,3,7]. However, increasing the work-function difference and modifying the barrier profile can simultaneously alter the tunneling current and diode resistance. Therefore, a meaningful evaluation of HfSiON requires a self-consistent analysis of the metal–dielectric–metal energy barriers and the resulting nonlinear current–voltage characteristics, rather than simply comparing dielectric constants or nominal material properties. Accordingly, the present work investigates an asymmetric Pt/HfSiON/Ti MIM tunnel diode operating at 28.3 THz, with the objective of assessing HfSiON as an alternative tunneling dielectric for LWIR rectenna applications. The device is analyzed using the full Simmons quantum-tunneling model implemented in MATLAB release 2025b. The analysis systematically evaluates the current density–voltage and current–voltage characteristics, zero-bias dynamic resistance, rectification asymmetry, nonlinearity, responsivity, and temperature dependence. Particular attention is given to the zero-bias operating regime because a passive infrared rectenna must rectify the small AC voltage generated by the receiving antenna without requiring an external DC bias [1,3,7]. The resulting electrical characteristics are then discussed in terms of their implications for high-frequency rectification and antenna–diode integration.

The research gap addressed by this study is therefore not simply the introduction of another dielectric material into an MIM structure. Rather, it is the lack of a systematic evaluation of HfSiON as an ultrathin tunneling barrier in an asymmetric MIM diode specifically targeted at 28.3 THz, including the simultaneous assessment of the electrical FOMs that determine its suitability for rectenna integration.

By combining a physically based tunneling model with a comprehensive analysis of resistance, asymmetry, nonlinearity, responsivity, and temperature dependence, this study aims to determine whether the Pt/HfSiON/Ti architecture can provide a competitive platform for next-generation LWIR rectification. Despite the extensive investigation of Al_2_O_3_, NiO, TiO_2_, ZnO, Ta_2_O_5_, Nb_2_O_5_, Sc_2_O_3_, and HfO_2_ based tunnel barriers, the application of HfSiON as the dielectric barrier of an asymmetric MIM rectifier specifically targeting 28.3 THz (10.6 μm) remains insufficiently explored in the rectenna literature. The remainder of this paper is organized as follows. Section 2 presents the operating principles of MIM tunnel diodes together with the theoretical formulation based on the Simmons tunneling model. Section 3 describes the design methodology and optimization procedure of the proposed Pt/HfSiON/Ti structure. Section 4 discusses the numerical results and evaluates the principal figures of merit governing the diode performance. Section 5 compares the proposed device with previously reported MIM rectifiers, while Section 6 summarizes the main conclusions and outlines future research directions.

## 2. MIM Tunnel Diodes: Operating Principles and Performance Metrics

**Figure 1:**
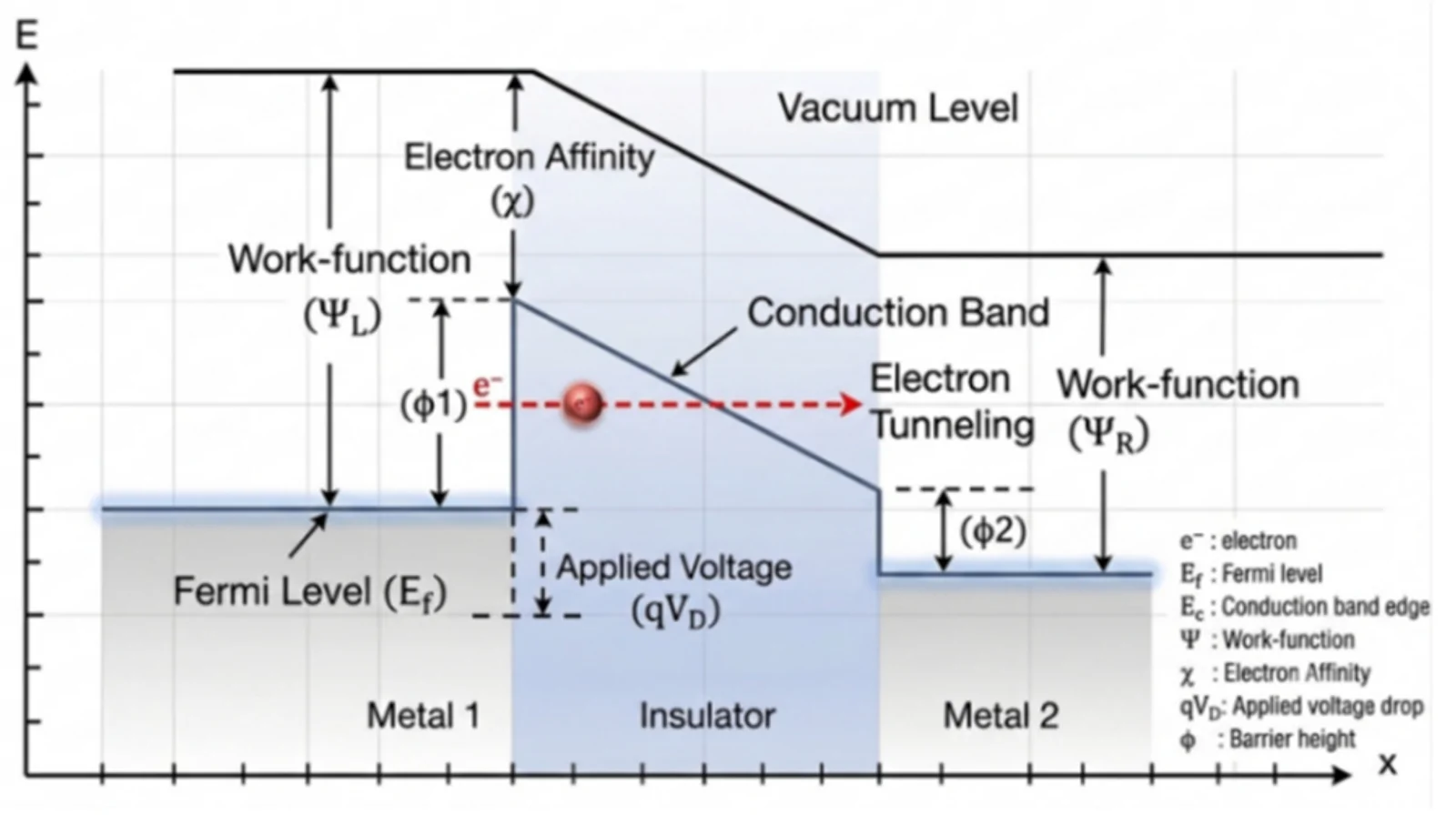
Energy-band profile of a MIM tunnel diode.

MIM tunnel diodes are nanoscale electronic devices consisting of two metallic electrodes separated by an ultrathin insulating layer, typically a few nanometers thick. MIM diodes can be classified as symmetric or asymmetric according to the electronic properties of the two metal–insulator interfaces. Symmetric MIM diodes employ identical electrode materials, resulting in similar barrier properties at both metal–insulator interfaces. In contrast, asymmetric diodes, as illustrated in Figure 1, employ different electrode materials with distinct work functions, leading to unequal barrier heights at the two interfaces. The latter configuration is the focus of the present work. Figure 1 illustrates the potential-energy profile across the dielectric, which is modeled as an asymmetric trapezoidal barrier, in contrast to the symmetric rectangular barrier expected for identical electrodes.

Barrier asymmetry is essential for rectification because it causes electron tunneling to depend on the polarity of the applied voltage. The tunneling current is highly sensitive to both the physical and electronic properties of the junction. In particular, the electron transmission probability depends exponentially on the barrier height and thickness and is further affected by the effective electron mass and the electric field established across the dielectric.

Therefore, the appropriate selection and combination of the two electrodes and the insulating material are critical for simultaneously controlling the magnitude of the tunneling current and the degree of rectification. Under different bias polarities (positive or negative), the applied voltage alters the tunneling-barrier profile differently, changing the barrier thickness and effective height experienced by electrons. This leads to different tunneling probabilities and, consequently, different current levels for the two bias polarities. The resulting asymmetric I–V characteristic enables the diode to rectify an applied AC signal, producing a net DC. In the present work, the tunneling current density is calculated using the Simmons quantum-mechanical tunneling model [16,17]. Within the adopted energy-band approximation, the two metal–insulator interfaces are characterized by different barrier heights determined by the relative alignment between the metal work functions and the electron affinity of the insulating material. In particular, the barrier height at each interface is approximated as the difference between the electrode work function and the electron affinity of the dielectric. Under an applied bias voltage, the asymmetric potential profile is modified according to both the magnitude and polarity of the applied voltage, resulting in a bias-dependent tunneling probability and current density, J(V). The Simmons model provides an analytical quantum-mechanical description of electron transport through ultrathin insulating barriers and is therefore employed to calculate the tunneling current density [16]:

(1) $$J(V)\;=\;\frac{q}{2\pi hd^{2}}\left[(\Phi \;-\;\frac{\mathrm{qV}}{2})e^{-\mathrm{Ad}\sqrt{\Phi -\frac{\mathrm{qV}}{2}}}-(\Phi \;+\;\frac{\mathrm{qV}}{2})e^{-\mathrm{Ad}\sqrt{\Phi +\frac{\mathrm{qV}}{2}}}\right]$$

where $A\;=\;\frac{4\pi \sqrt{2m}}{h}$; q is the electric charge; h is Planck’s constant; V is the applied bias; Φ  is the barrier height of the electrode–insulator interface from which electrons are tunneling, given such as the difference between the work function of the metal and the electron affinity of the oxide; m is the effective electron mass; d is the tunnel barrier thickness; and J is the tunneling-current density. The corresponding total tunneling current is obtained from

I(V) = J(V) × A,(2)

where A is the electrically active overlap area of the MIM junction [16]. This formulation allows the electrical response of the junction to be directly related to the material-dependent barrier properties and the nanoscale geometry of the device. The rectification performance of the investigated MIM junctions is subsequently evaluated using a consistent set of FOM extracted from the calculated I–V characteristics, including the zero-bias differential resistance, asymmetry, nonlinearity factor, and responsivity, as summarized in Table 1.

The parameters reported in Table 1 describe complementary aspects of the tunneling and rectification behavior and must therefore be considered as distinct quantities. In a nonlinear tunneling junction, the DC resistance and the differential resistance represent different physical quantities and generally depend on the operating point. The static resistance is defined as R_DC_ = V/I, whereas the differential or dynamic resistance is given by R_diff_ = (dI/dV)^−1^. For a zero-DC-bias MIM diode operating in the small-signal regime, the differential resistance is determined locally from the slope of the DC I–V characteristic at V = 0: R_D0_ = [dI/dV]^−1^_V = 0_.

For sufficiently small AC excitation, it can be experimentally approximated as R_D0_ ≈ V_AC_/I_AC_. However, when the AC amplitude is sufficiently large to probe the nonlinear I–V response, this ratio represents a finite-signal dynamic impedance rather than the infinitesimal differential resistance. Therefore, the two quantities should be distinguished, particularly for strongly nonlinear MIM junctions [17].

The asymmetry quantifies the difference between forward I_F_ and reverse I_R_ tunneling currents at finite bias and is defined as f_ASYM_ = |I_F_(+V)|/|I_R_(−V)|. Accordingly, f_ASYM_= 1 corresponds to a symmetric I–V characteristic, whereas values significantly different from unity indicate asymmetric carrier transport and rectification behavior. The nonlinearity, f_NL_, characterizes the deviation of the I–V response from its local linear behavior. A value of f_NL_ approaching unity in the immediate vicinity of zero bias should not be interpreted as evidence that the junction is incapable of rectification. The zero-bias responsivity characterizes the junction’s capability to rectify an AC excitation in the absence of an externally applied DC voltage. Together, these parameters establish a consistent framework for evaluating the material-dependent tunneling behavior, rectification capability, and high-frequency suitability of the proposed MIM junction for infrared energy-harvesting applications. It is important to distinguish between strict zero-bias equilibrium and zero-DC-bias rectification. At thermodynamic equilibrium, when both the DC and AC excitations are absent (V_DC_ = 0 and V_AC_ = 0), an asymmetric MIM junction cannot sustain a net DC, as required by detailed balance. Therefore, electrode and barrier asymmetry alone does not produce an equilibrium current. In contrast, under AC excitation (V_AC_ ≠ 0), the condition V_DC_ = 0 corresponds to zero-DC-bias operation, allowing the nonlinear junction to rectify the incident AC signal and generate a net DC. Under AC excitation, the incident electromagnetic field periodically modulates the tunneling barrier, resulting in a nonlinear tunneling current that differs for positive and negative voltage excursions due to the asymmetric barrier heights at the two metal–insulator interfaces. Consequently, the nonlinear and asymmetric I–V characteristic produces a non-zero time-averaged current, I_DC_, which constitutes the rectified DC output. This mechanism provides the physical basis for zero-DC-bias rectification in MIM diodes, enabling electromagnetic energy harvesting without an external DC voltage. The zero-bias responsivity is therefore a key parameter for evaluating the ability of the nonlinear junction to convert an incident AC excitation into a rectified DC output [17]. The high-frequency performance is governed by the RC time constant of the junction, with the cutoff frequency given by fc = 1/2πR_D_C_D_, where R_D_ is the relevant diode resistance and the junction capacitance is determined by C_D_ = ε_0_ε_r_A/d. Thus, reducing the active junction area decreases the capacitance and increases the RC-limited bandwidth, while reducing the dielectric thickness can enhance the tunneling current [18]. These parameters must, however, be optimized to maintain reliable electrical isolation, stable barrier properties, and sufficient tunneling current. The simultaneous optimization of barrier thickness, active area, dielectric properties, and tunneling resistance is essential for balancing rectification performance and high-frequency operation. Based on these considerations, an asymmetric Pt/HfSiON/Ti MIM tunnel diode is proposed for LWIR operation at 28.3 THz. The different work functions of Pt (≈5.65 eV) and Ti (≈4.33 eV) establish dissimilar metal–insulator interfaces and, consequently, an asymmetric tunneling potential, with a lower barrier expected at the Ti/HfSiON interface and a higher barrier at the Pt/HfSiON interface. HfSiON is selected for its favorable electronic properties, thermal stability, dielectric reliability, and compatibility with nanoscale fabrication. The junction features an active area of approximately 21 × 21 nm^2^ and a 2 nm-thick dielectric layer, providing a suitable compromise between high tunneling current density and low junction capacitance. The target frequency of 28.3 THz is determined by the electromagnetic resonance of the receiving antenna, while the tunneling model describes the nonlinear transport and rectification behavior of the MIM junction. The junction geometry and dielectric properties are then used to determine the junction capacitance, and the resulting electrical parameters are incorporated into a simplified antenna–diode equivalent circuit to assess the high-frequency compatibility of the integrated system. Accordingly, the high-frequency assessment follows three complementary steps: (i) calculation of the nonlinear MIM tunneling characteristics to establish the rectification mechanism; (ii) determination of the junction capacitance from the MIM geometry and dielectric properties; and (iii) incorporation of the relevant junction and antenna parameters into an equivalent-circuit RC analysis to evaluate the electrical compatibility of the integrated rectenna with the target 28.3 THz operation. The relevant material, geometrical, and electrical parameters used to evaluate the proposed Pt/HfSiON/Ti MIM diode and its estimated high-frequency performance are summarized in Table 2 [10,12,13,14].

## 3. Design and Performance Optimization of the Proposed MIM Diode

**Figure 2:**
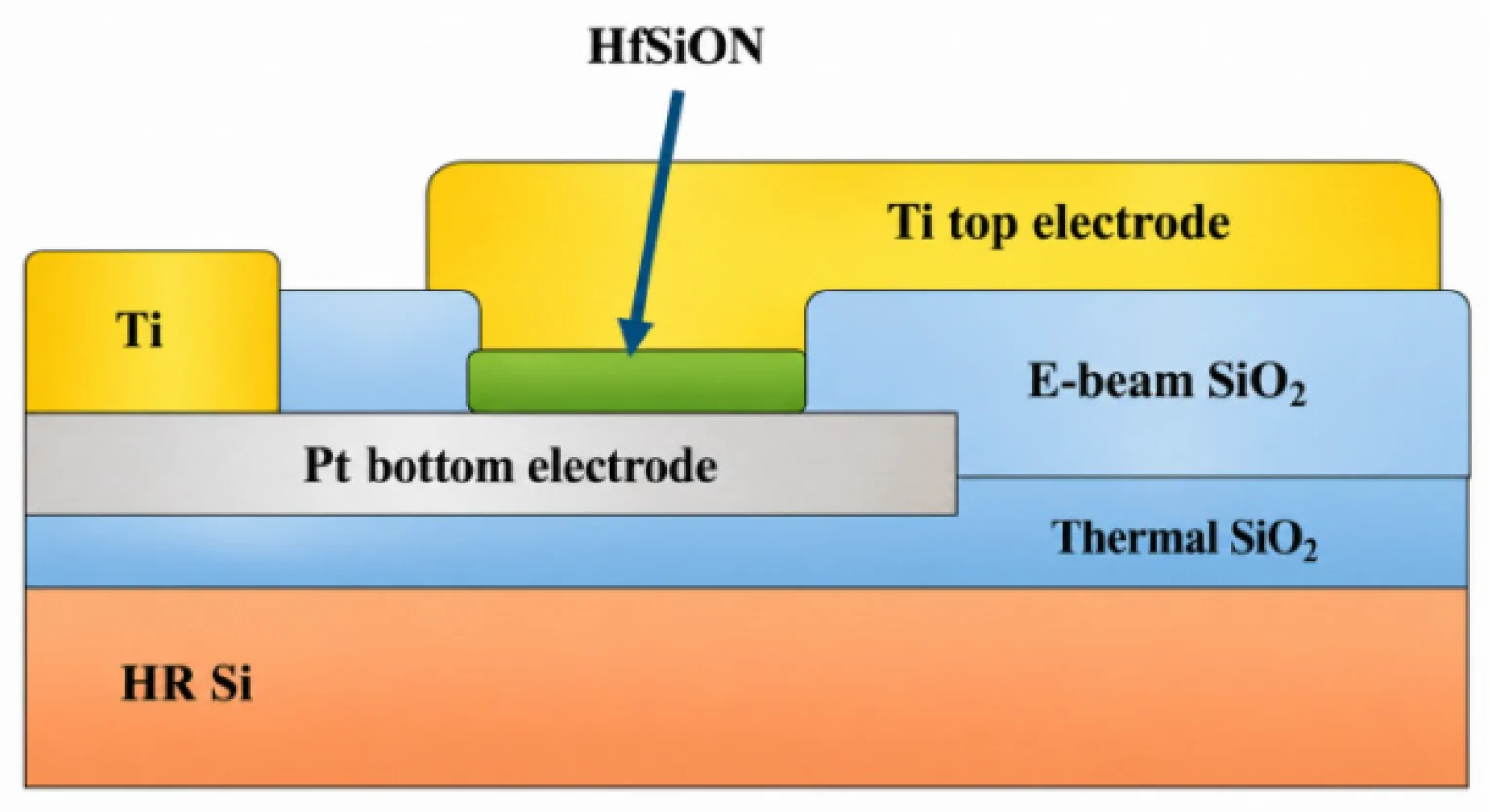
Schematic cross-section of the Pt-HfSiON-Ti MIM Diode.

As shown in Figure 2, the proposed MIM diode adopts a multilayer architecture designed for efficient operation at 28.3 THz. From bottom to top, the structure comprises a 50 nm-thick gold (Au) reflector, which acts as a ground plane and enhances electromagnetic confinement and constructive interference while reducing radiation losses toward the substrate. The reflector is supported by a 500 nm-thick high-resistivity Si substrate (ε_r_ = 11.9, ρ = 10,000 Ωcm). A 300 nm-thick SiO layer (ε_r_ = 3.9, tanδ = 0.001) is introduced above the substrate to improve impedance matching and enhance electromagnetic coupling between the receiving antenna and the rectifying junction. A 3 nm-thick Cr layer is subsequently employed as an adhesion layer to ensure reliable integration of the upper metallic contacts [19,20]. The active rectifying element consists of an asymmetric Pt/HfSiON/Ti MIM junction, in which 100 nm-thick Pt and Ti electrodes are separated by a 2 nm-thick HfSiON insulating barrier. The difference in the electrode work functions establishes an asymmetric tunneling potential, while the ultrathin dielectric barrier facilitates quantum-mechanical electron tunneling and enhances the nonlinear response required for zero-bias rectification. The junction area is restricted to 21 × 21 nm^2^ (441 nm^2^) to minimize parasitic capacitance while maintaining strong electric-field confinement and a sufficiently high tunneling current density. The main geometrical, material, and electrical parameters of the proposed structure are summarized in Table 3 [9,10,13,14,15].

The choice of the electrode–dielectric interfaces plays a decisive role in determining the tunneling characteristics and rectification capability of the proposed asymmetric MIM junction. The Pt/HfSiON/Ti junction exhibits a pronounced asymmetry in the tunneling barrier, with barrier heights of 0.33 eV and 1.65 eV at the Ti/HfSiON and Pt/HfSiON interfaces, respectively.

**Figure 3:**
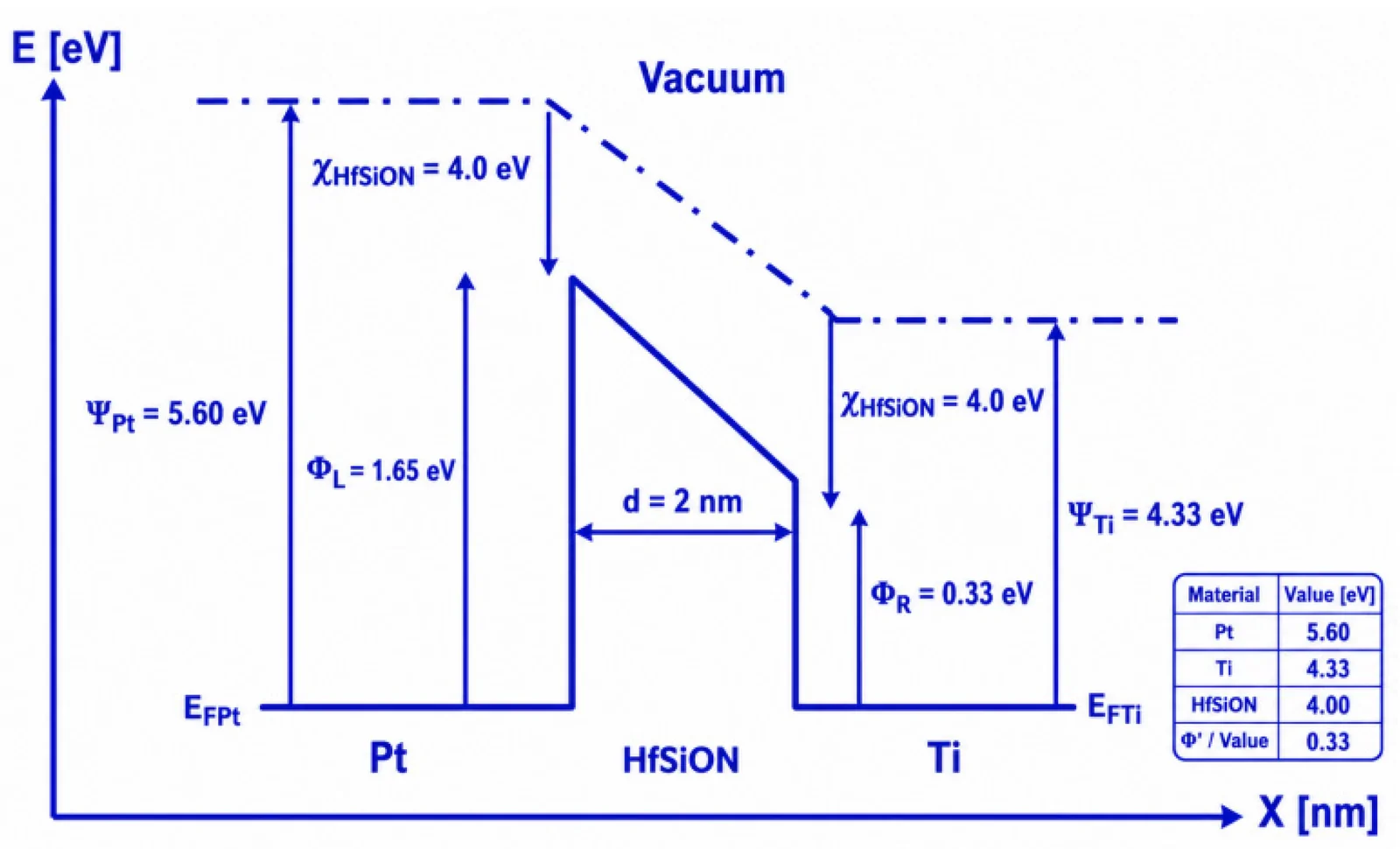
Energy diagram of the Pt/HfSiON/Ti MIM diode shows a triangular barrier model (Φ = Ψmetal − χinsulator).

The substantially lower barrier on the Ti side favors electron transmission through the 2 nm HfSiON layer, whereas the higher Pt-side barrier limits tunneling for the opposite voltage polarity. As a result, reversing the applied voltage produces substantially different tunneling conditions across the junction, giving rise to the asymmetric potential profile shown in Figure 3 and, consequently, to the polarity-dependent transport response required for rectification. The asymmetric tunneling barrier is central to the rectifying behavior of the Pt/HfSiON/Ti MIM junction. Under opposite voltage polarities, the potential profile is modified differently, resulting in preferential electron tunneling through the lower-barrier Ti/HfSiON interface under forward bias, while the higher barrier at the Pt/HfSiON interface substantially reduces, but does not completely suppress, reverse tunneling. This polarity-dependent difference in transmission probability produces the asymmetric and nonlinear I–V response required for rectification.

HfSiON provides a favorable combination of electronic and dielectric properties for this architecture. Its electron affinity results in a relatively low tunneling barrier at the Ti/HfSiON interface, promoting electron transmission, while its moderate dielectric permittivity helps limit the junction capacitance and associated RC losses at high frequency. The calculated energy-band diagram in Figure 3, based on the material parameters reported in Table 3, shows barrier heights of 0.33 eV and 1.65 eV at the Ti/HfSiON and Pt/HfSiON interfaces, respectively. This pronounced barrier asymmetry establishes the unequal tunneling probabilities required for rectification. Under AC excitation, the resulting nonlinear transport favors conduction during one half-cycle and suppresses it during the opposite half-cycle, thereby generating a net DC component and providing the physical basis for zero-DC-bias AC-to-DC conversion in the proposed LWIR rectenna [21,22].

**Figure 4:**
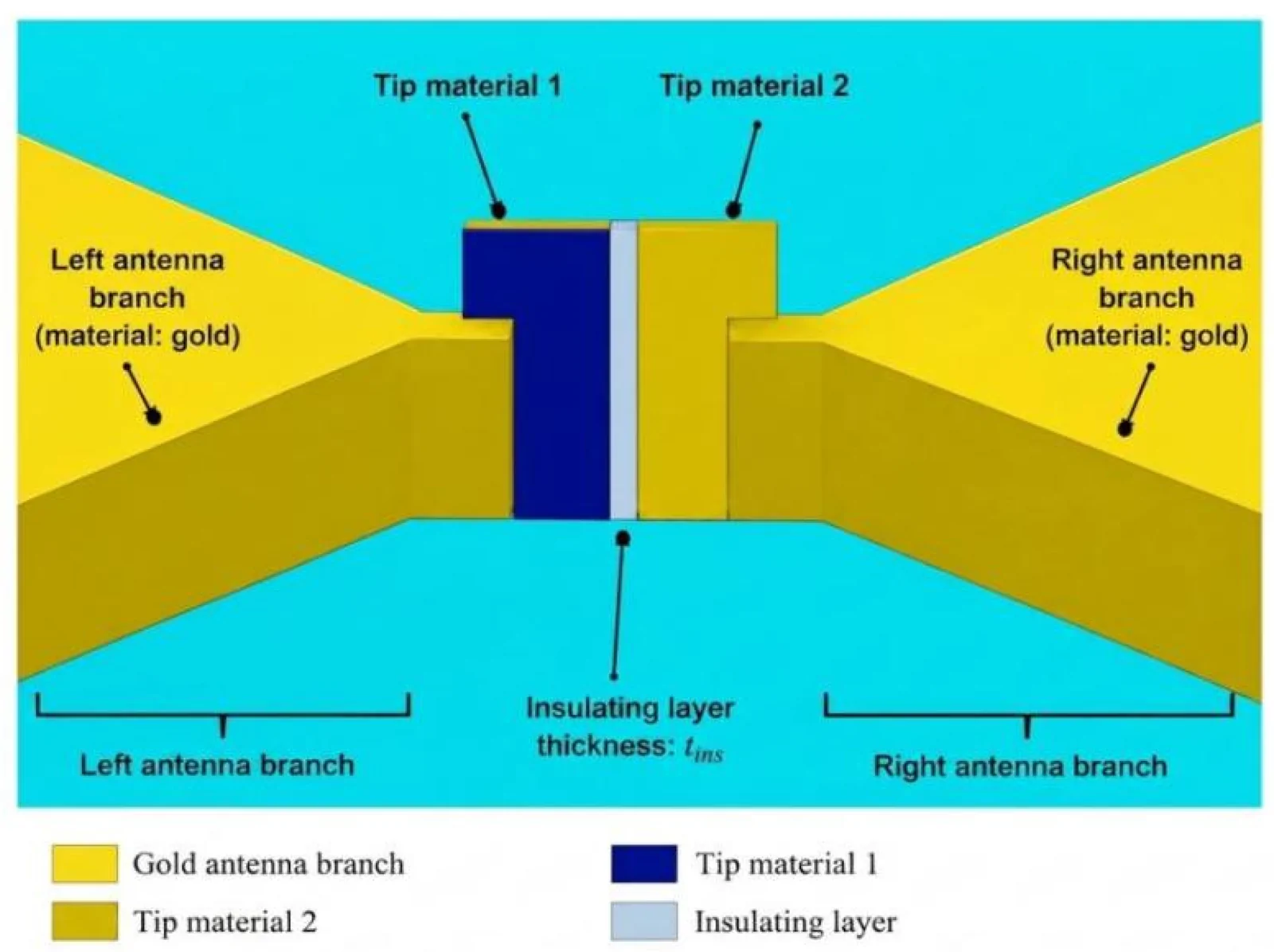
Detail of an asymmetric MIM diode integrated with an antenna structure.

Figure 4 illustrates a detailed view of the asymmetric MIM diode integrated with the receiving antenna. The device consists of two opposing antenna arms that gradually taper toward a central nanogap, forming the two metallic electrodes of the MIM junction. The antenna arms are made of gold and serve both as electromagnetic receiving elements and as electrical contacts to the MIM diode. At the center of the structure, the two metallic electrodes terminate in different metal materials, forming an asymmetric MIM configuration. A thin insulating layer is positioned between the two tips, defining the tunneling barrier with a thickness of tins. The insulating layer electrically separates the two electrodes while enabling electron transport through quantum tunneling. The resulting structure can be represented as Metal 1/Insulator/Metal 2, where the two metals may have different work functions.

This asymmetric electrode configuration enables control of the tunneling barrier heights and promotes the nonlinear and rectifying response of the junction. The antenna arms concentrate the incident electromagnetic field at the central nanogap, generating a high-frequency voltage across the insulating barrier. Consequently, the antenna and MIM junction are strongly electromagnetically and electrically coupled, enabling the structure to function as a rectifying element for LWIR radiation [15].

## 4. Electrical Parameters and FOM Analysis of the Pt–HfSiON–Ti MIM Diode

### 4.1. Analysis of Asymmetric Current–Voltage (I–V) Response

The rectification behavior of the proposed MIM diode is governed by quantum-mechanical electron tunneling through an asymmetric potential barrier arising from the dissimilar electronic properties of the two metal–insulator interfaces. The forward and reverse currents extracted from the calculated I–V characteristics exhibit a pronounced exponential dependence on the applied diode voltage, which can be expressed as I_F_(V_D_) = f_ASYM_e^αVD^ and I_R_(V_D_) = −e^−αVD^, respectively. Here, V_D_ denotes the applied diode voltage, while α characterizes the voltage dependence of the tunneling current. The rectification performance is quantified by the asymmetry, f_ASYM_ = 10^3.3137(ΨM1−ΨM2)^, which depends strongly on the work-function difference between the two electrodes [23,24]. This expression represents the empirical/model-dependent relationship adopted in the present analysis and should therefore not be regarded as a universal definition of the asymmetry for MIM diodes. For the Pt/HfSiON/Ti junction, the work-function difference of ΔΨ = 1.33 eV produces a pronounced asymmetry in the tunneling barriers and results in f_ASYM_ ≈ 2.5 × 10^4^. This large asymmetry reflects the substantial imbalance between forward and reverse tunneling currents and, consequently, the strong rectification capability of the proposed MIM diode. In general, increasing the electrode work-function difference enhances the asymmetry of the interfacial barriers, promoting tunneling under one polarity while suppressing carrier transmission under the opposite polarity.

Consequently, the electron transmission probability becomes strongly bias-dependent: under forward bias, the reduced effective barrier facilitates tunneling and promotes a larger current, whereas under reverse bias, the higher barrier suppresses electron transmission and limits the reverse current. This asymmetric tunneling process ultimately gives rise to the pronounced rectifying behavior of the device, demonstrating that charge transport is primarily determined by the relative barrier heights at the two metal–HfSiON interfaces [25].

### 4.2. Electrical Response of the Asymmetric MIM Diode: Simulation of J–V and I–V Characteristics

Numerical simulations were carried out in MATLAB release 2025b using the Simmons tunneling formalism described by Equation (1) to investigate the current-density–voltage (J–V) and current–voltage (I–V) characteristics of the proposed asymmetric Pt/HfSiON/Ti MIM diode. The applied voltage was varied from −0.5 to +0.5 V, while the HfSiON thickness was fixed at 2 nm. Such an ultrathin dielectric layer provides a tunneling regime in which direct quantum-mechanical electron tunneling is expected to dominate the charge-transport process.

**Figure 5:**
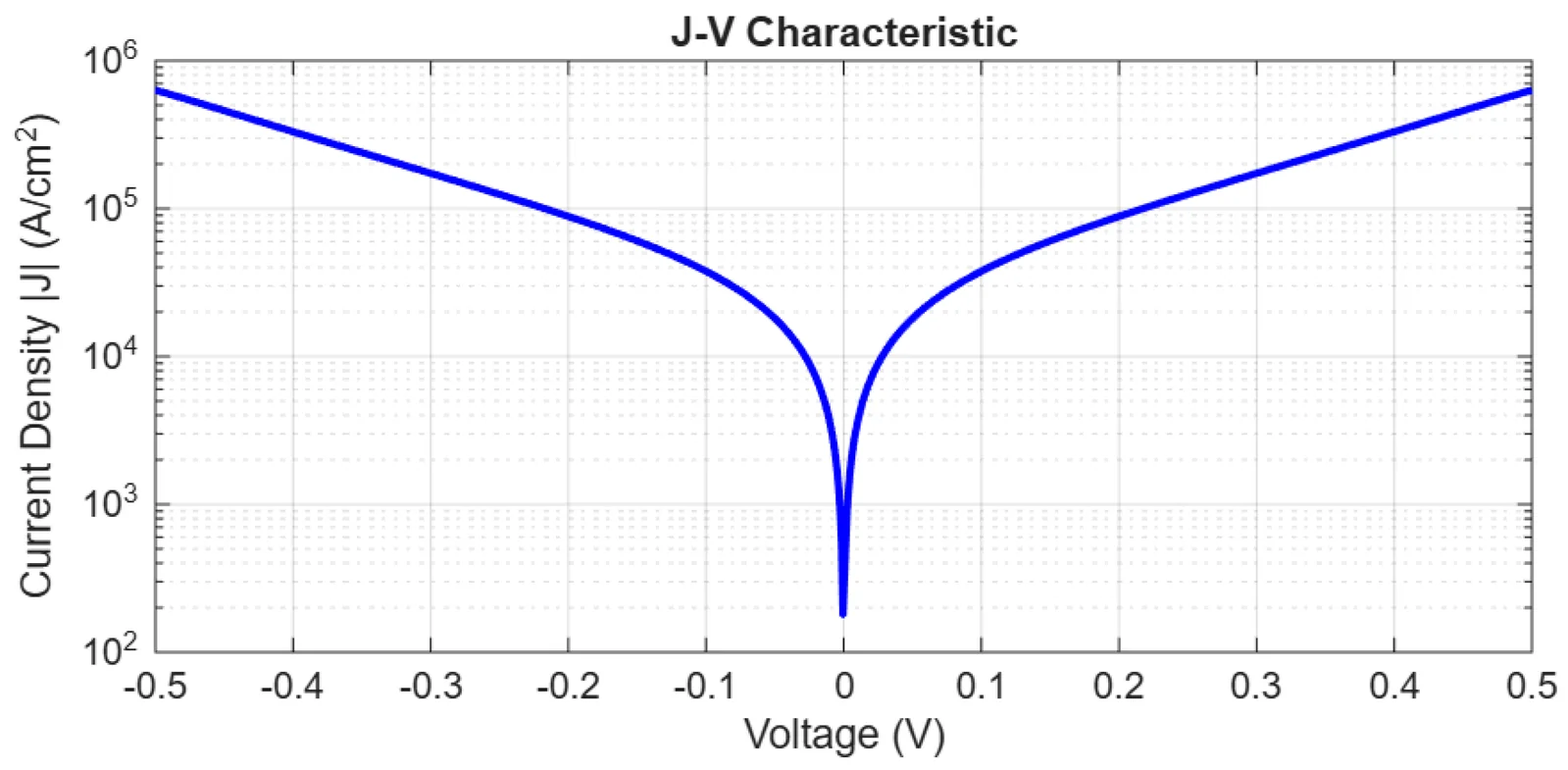
Logarithmic current density–voltage (J–V) characteristic of the Pt/HfSiON/Ti MIM diode.

**Figure 6:**
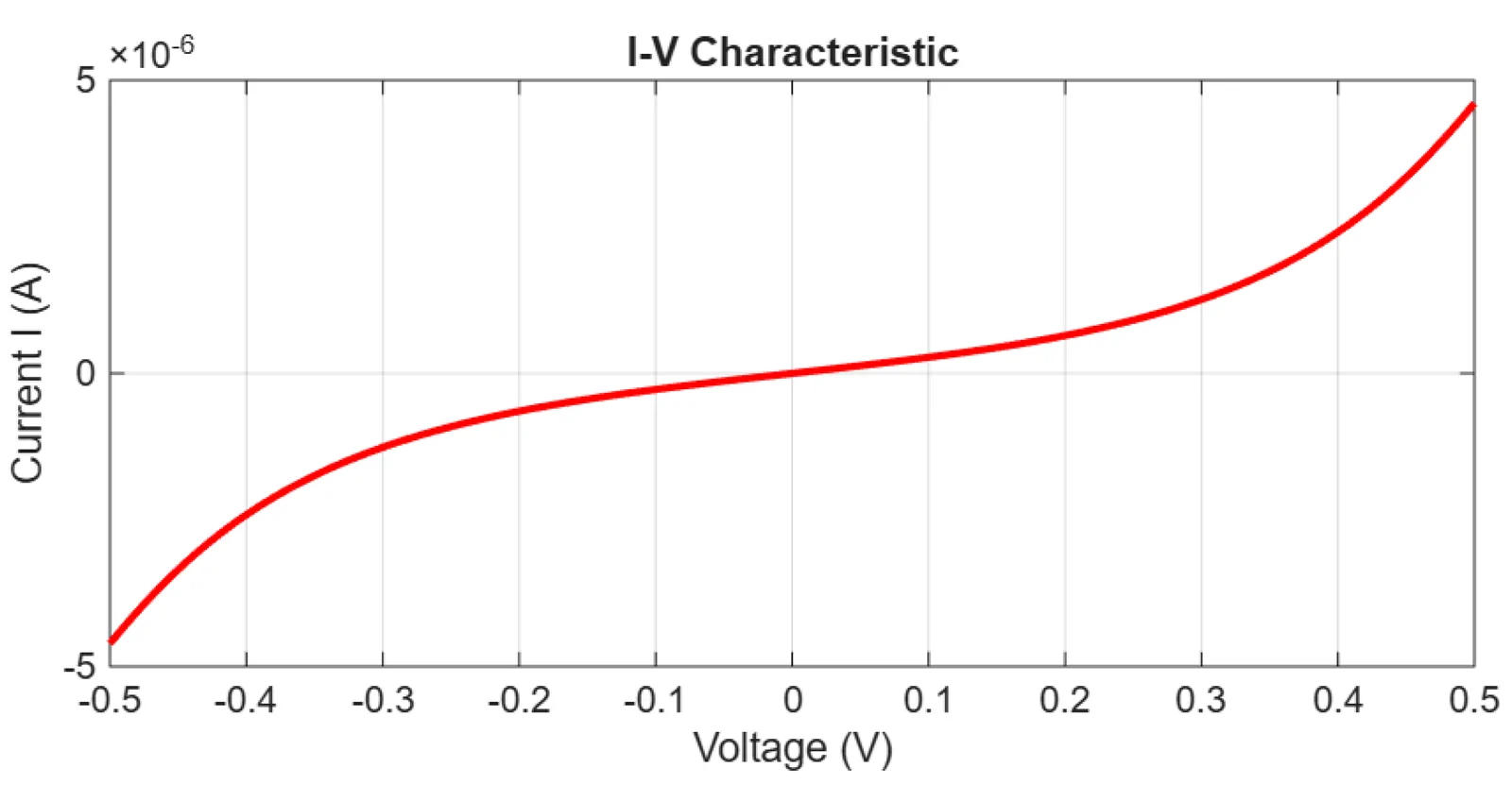
Simulated current–voltage (I–V) characteristic of the Pt/HfSiON/Ti MIM diode.

The simulated semi-logarithmic J–V characteristic shown in Figure 5 exhibits the strong voltage dependence associated with tunneling through the ultrathin insulating barrier. For a junction area of 21 × 21 nm^2^ (441 nm^2^), the calculated current density reaches approximately 1.78 × 10^2^ A/cm^2^ under zero-DC-bias AC excitation and increases to 6.32 × 10^5^ A/cm^2^ at a voltage amplitude of ±0.5 V. These results demonstrate the ability of the ultrathin HfSiON barrier to support high-density electron tunneling within a highly compact junction. The corresponding I–V characteristic in Figure 6 shows pronounced rectification, resulting from unequal tunneling barriers at the Pt/HfSiON and Ti/HfSiON interfaces. Under forward operation, the current reaches approximately 4.61 × 10^−6^ A, whereas the reverse current is limited to approximately 1.84 × 10^−10^ A, yielding a forward-to-reverse current ratio of approximately 2.5 × 10^4^. This pronounced current asymmetry confirms the strong rectifying response of the junction and highlights the role of electrode-induced barrier asymmetry in governing charge transport. Under AC excitation, a rectified zero-DC-bias current of approximately 1.30 × 10^−9^ A is obtained. This result is particularly relevant to the proposed rectenna architecture, as it demonstrates the generation of a net DC response from an AC excitation without the need for an externally applied DC bias. This zero-DC-bias response is particularly relevant to rectenna applications, where efficient nonlinear transport around the zero-bias operating point enables the conversion of weak, high-frequency electromagnetic excitation into a rectified DC output. The pronounced difference between forward and reverse conduction originates from the unequal effective tunneling barriers associated with the dissimilar Pt and Ti electrodes. Under one voltage polarity, the barrier profile favors electron transmission and results in a higher tunneling current, whereas the opposite polarity produces a substantially lower transmission probability and, consequently, a strongly suppressed current. This polarity-dependent modulation of the tunneling probability provides the physical basis for the observed rectification and is consistent with the operating principles reported for asymmetric MIM tunnel diodes [26]. Overall, the numerical results indicate that the Pt/HfSiON/Ti junction combines an ultrathin dielectric barrier with efficient direct quantum tunneling and pronounced electrode-induced barrier asymmetry. The resulting high current density, large forward-to-reverse current contrast, and zero-DC-bias rectified response demonstrate the potential of the proposed MIM junction for high-frequency rectification and LWIR rectenna applications [26,27,28,29,30,31].

### 4.3. Effect of Temperature on the I–V Characteristics of the MIM Tunnel Diode

**Figure 7:**
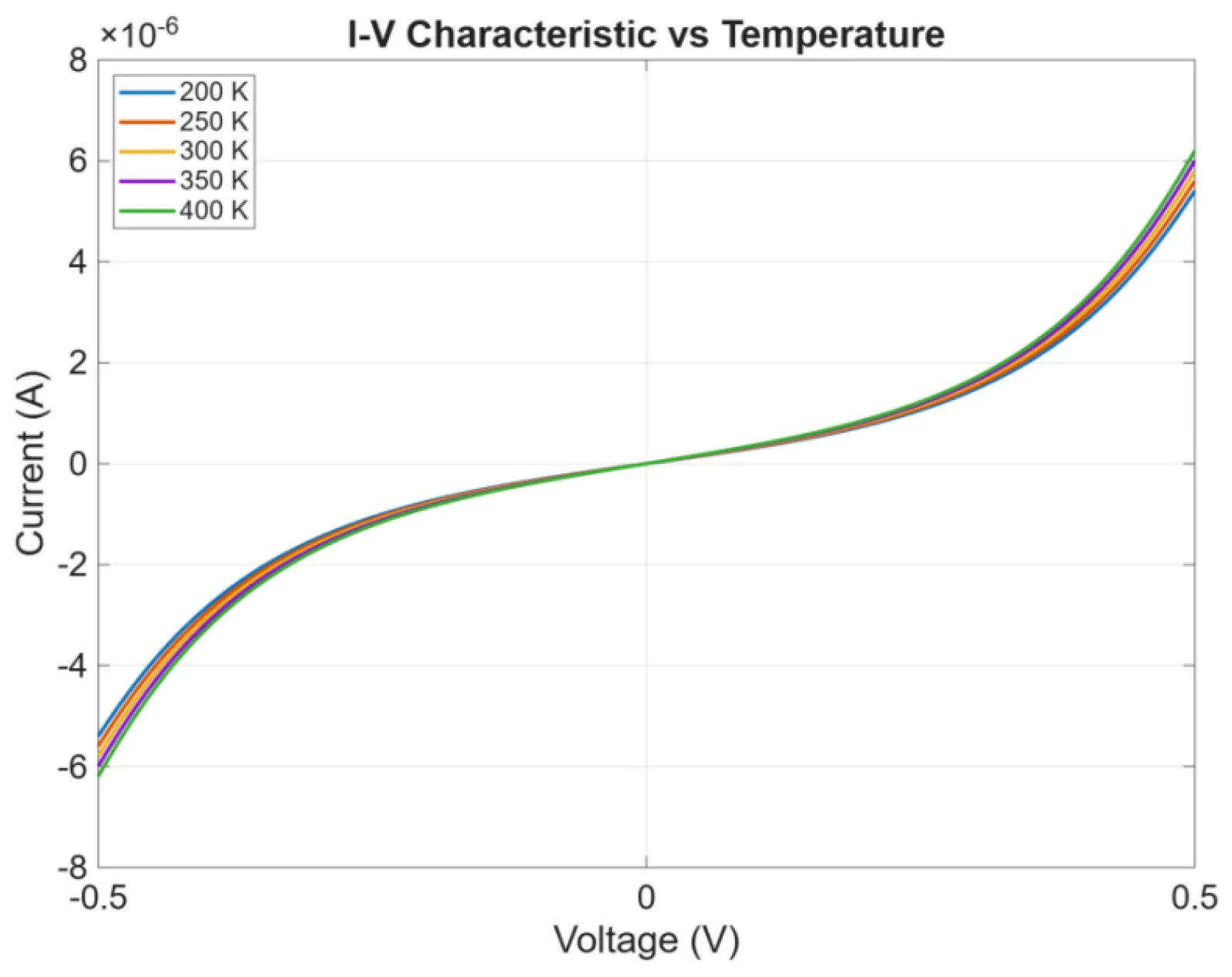
I–V Characteristic vs. Temperature. Semi-logarithmic plot of current I versus voltage (V) across temperatures ranging from 200 to 400 K. The values on both axes have been enlarged for enhanced legibility.

The possible temperature-dependent electrical characteristics of the MIM tunnel diode were investigated using the full Simmons tunneling model over a voltage range of ±0.5 V and at temperatures between 200 and 400 K. The resulting current density (J) is given by [16]:

(3) $$J\;=\;\frac{q}{2\pi hd^{2}}\left[\phi_{1}\exp(-\frac{2d\sqrt{2m\phi_{1}}}{\hbar })\;-\;{\;\phi }_{2}\exp(-\frac{2d\sqrt{2m\phi_{2}}}{\hbar })\right]$$

where $\phi_{1}\;=\;\phi \;-\;\frac{\mathrm{qV}}{2}$ and $\phi_{2}\;=\;\phi \;+\;\frac{\mathrm{qV}}{2}\;$ represent the effective barrier heights modified by the applied voltage V, d  is the insulator thickness, m  is the electron mass, and ℏ is the reduced Planck constant. To account for temperature effects, a simplified thermal correction factor was introduced to model Fermi–Dirac broadening:

(4) $$I\;=\;A\cdot J\cdot \left(1\;+\;\frac{k_{B}T}{qV_{T}}\right)$$

where I is the current, A  is the MIM area, $k_{B}$ is the Boltzmann constant, T  is the temperature, and $V_{T}\;$ is a reference thermal voltage parameter. The simulated I–V curves in Figure 7 exhibit a modest increase in current magnitude as the temperature increases, while maintaining the overall shape and rectifying behavior of the MIM diode.

To evaluate the influence of temperature on the transport properties of the proposed MIM junction, the electrical response was simulated over the range of 200–400 K using the Simmons tunneling model. As shown in Figure 7, the semi-logarithmic I–V characteristics exhibit a progressive increase in current density over the investigated bias range from −0.5 to +0.5 V as the temperature is increased. This behavior can be attributed primarily to the increased thermal population of electronic states and the broadening of the electron energy distribution, which enhance the availability of carriers with sufficient energy to contribute to transport across the ultrathin HfSiON barrier. Under applied bias, barrier modulation further facilitates electron transmission by effectively reducing the tunneling barrier encountered by the carriers.

Despite this temperature-dependent increase in current, direct quantum-mechanical tunneling remains the dominant transport mechanism over the investigated temperature and voltage ranges, with thermal excitation providing an additional thermally assisted contribution. Unlike thermally activated transport mechanisms, quantum-mechanical tunneling is primarily determined by the properties of the potential barrier, including its height, thickness, asymmetry, and the electric field across the dielectric. Consequently, the intrinsic tunneling process is expected to exhibit only a limited dependence on temperature. However, if thermal excitation enhances carrier transport for both voltage polarities, the increase in temperature also partially reduces the intrinsic current asymmetry produced by the different Pt/HfSiON and Ti/HfSiON barrier heights. Consequently, while the absolute tunneling current increases with temperature, the rectification asymmetry evaluated at ±0.2 V exhibits a slight reduction.

This behavior highlights an important trade-off in the design of high-frequency MIM rectifiers: increasing temperature can enhance the overall tunneling current, but it may simultaneously increase undesired reverse-bias transport and reduce directional current selectivity. Therefore, maintaining a sufficiently asymmetric energy-barrier profile while limiting thermally assisted transport is essential for preserving strong rectification over a broad operating-temperature range.

**Figure 8:**
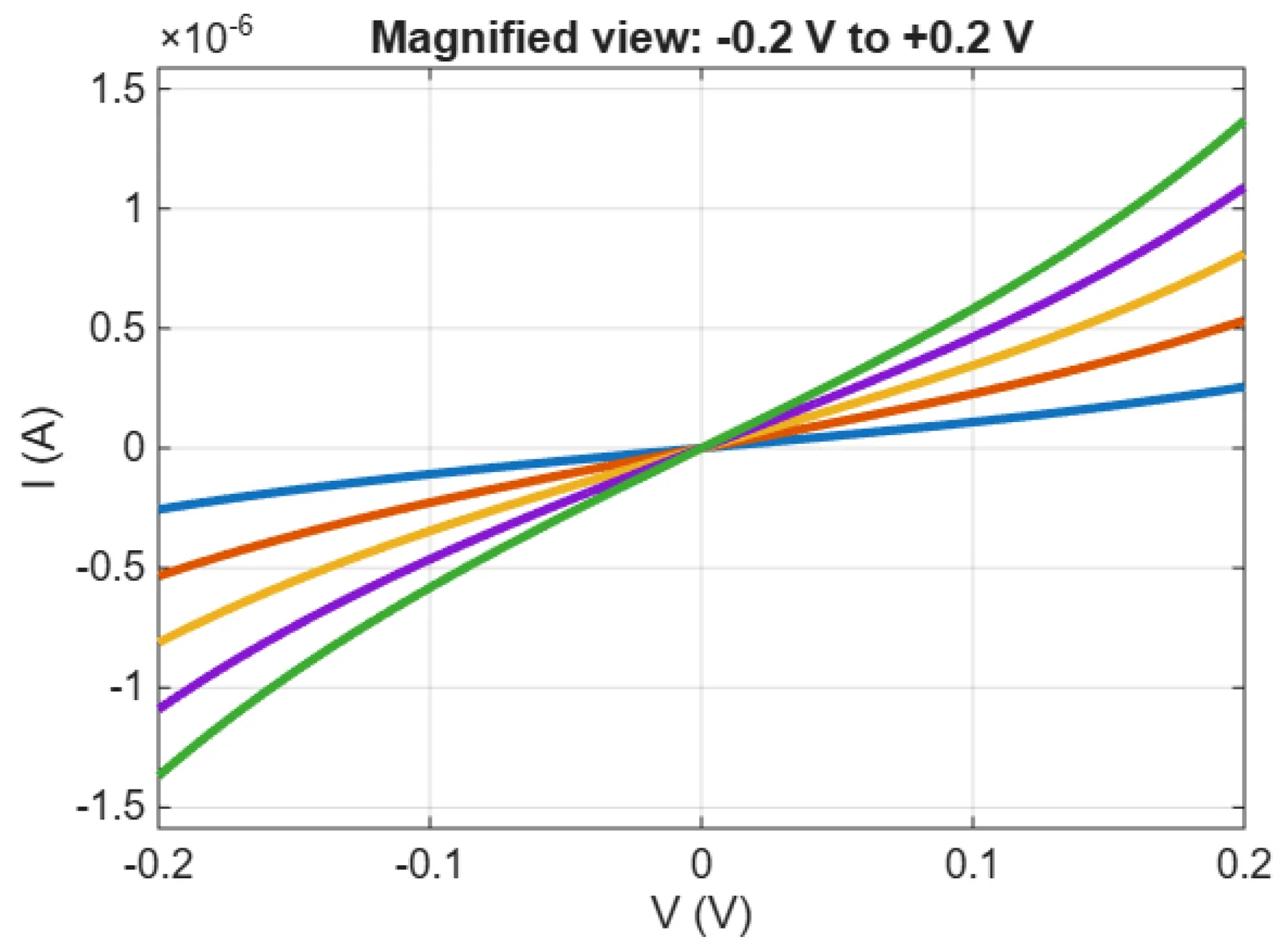
I–V Characteristics and Low-Bias Inset Behavior. Linear I–V characteristics of the MIM diode at different temperatures (main panel) alongside a magnified view (times 10 separation factor) of the low-bias region between −0.2 V and +0.2 V (inset panel). Tick values on the primary and inset axes are scaled up for optimal visual clarity.

Figure 8 presents the I–V characteristics, along with an inset providing an enlarged view of the low-bias region from −0.2 to +0.2 V. To better resolve the subtle temperature-dependent variations near zero bias, the deviation of each curve from the 300 K reference characteristic is magnified by a factor of 10. This enlarged representation clearly reveals the onset of thermally induced modulation of the electrical response even under low electric-field conditions.

In this work, the temperature analysis is therefore aimed primarily at assessing the thermal stability of the rectification response rather than demonstrating a strong temperature dependence of the tunneling probability. Within the investigated temperature range, the calculated rectified DC current I_DC_ shows only minor variations, indicating that the tunneling-dominated rectification mechanism remains substantially stable under the considered thermal conditions. At finite temperature, a rigorous description of the tunneling current requires the integration of the energy-dependent transmission probability over the Fermi–Dirac distributions of the two electrodes, thereby accounting explicitly for thermal broadening.

In the present tunneling-dominated regime, however, this broadening produces only a limited modification of the calculated transport response. The temperature-dependent correction adopted here should therefore be regarded as a first-order sensitivity analysis rather than as a complete finite-temperature quantum-transport model.

The limited variation in I_DC_ also suggests that the corresponding rectified output voltage V_DC_ is expected to remain relatively stable over the investigated temperature range. This thermal robustness is particularly advantageous for rectenna applications, where stable rectification under varying environmental conditions is essential for reliable energy harvesting. Overall, the results indicate that the nonlinear tunneling mechanism and the associated rectified response are only weakly sensitive to temperature within the considered range, supporting the potential of the proposed Pt/HfSiON/Ti MIM architecture for robust high-frequency LWIR energy-harvesting applications.

### 4.4. Dynamic Resistance

**Figure 9:**
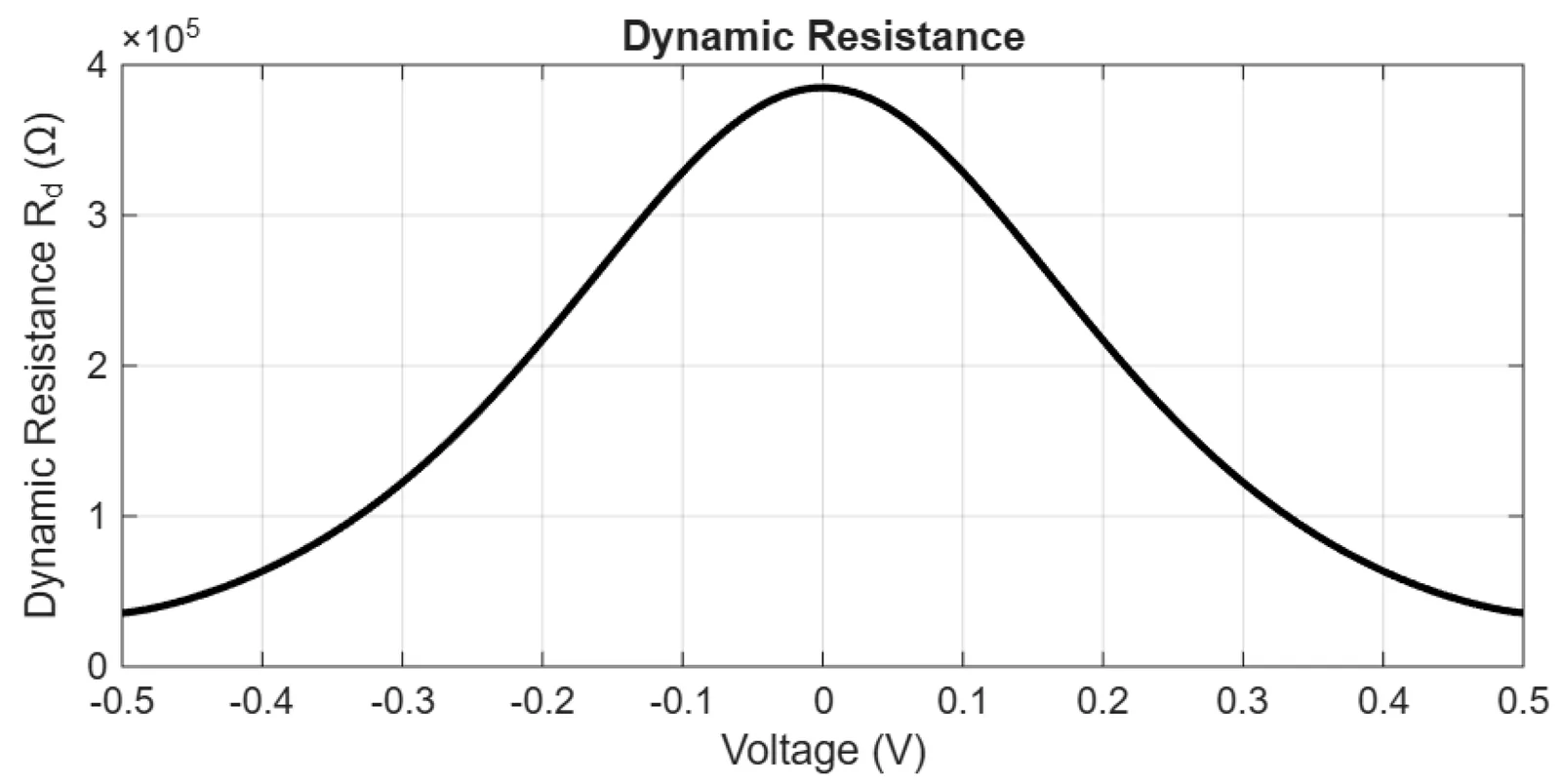
Dynamic resistance versus voltage curve of the Pt/HfSiON/Ti MIM tunnel junction diode.

Figure 9 presents the dynamic or differential resistance of the proposed Pt/HfSiON/Ti MIM tunnel diode as a function of the applied bias voltage.

At zero bias, the diode exhibits a differential resistance (R_D0_) of approximately (3.85 × 10^5^ Ω), the zero-bias differential resistance provides a meaningful measure of the local electrical impedance experienced by the rectifier under weak-signal operation and is therefore a key parameter for evaluating antenna–diode impedance matching and low-power rectification performance. As the magnitude of the applied bias increases, the differential resistance decreases markedly, reaching approximately 1.2 × 10^5^ Ω at ±0.3 V and 3.5 × 10^4^ Ω at ±0.5 V. This pronounced reduction originates from the strong bias dependence of quantum-mechanical tunneling in the ultrathin HfSiON barrier. The applied electric field modifies the effective tunneling barrier by reducing its width and altering its potential profile, thereby increasing the transmission probability and producing a steeper I–V response. Such field-assisted barrier modulation is a characteristic feature of MIM tunnel junctions and is responsible for their strongly nonlinear electrical response. In this regime, the nonlinear conductance around zero voltage governs the diode response to the small AC voltage generated by the receiving antenna. A suitable balance between zero-bias resistance, nonlinearity, asymmetry, and junction capacitance is therefore essential for maximizing the IR-to-DC conversion efficiency while minimizing impedance mismatch and parasitic losses. The extracted (R_D0_) [17] is higher than the typical input resistance of infrared nanoantennas, which is on the order of a few hundred ohms, indicating that direct impedance matching remains challenging.

For self-powered energy-harvesting systems, optimization should not be based solely on achieving a low resistance; rather, the zero-bias differential resistance must be considered together with the diode nonlinearity, rectification asymmetry, junction capacitance, and antenna impedance to maximize electromagnetic-to-electrical energy conversion at the target frequency [17].

### 4.5. Asymmetry $\left(f_{\mathrm{ASYM}}\right)$

**Figure 10:**
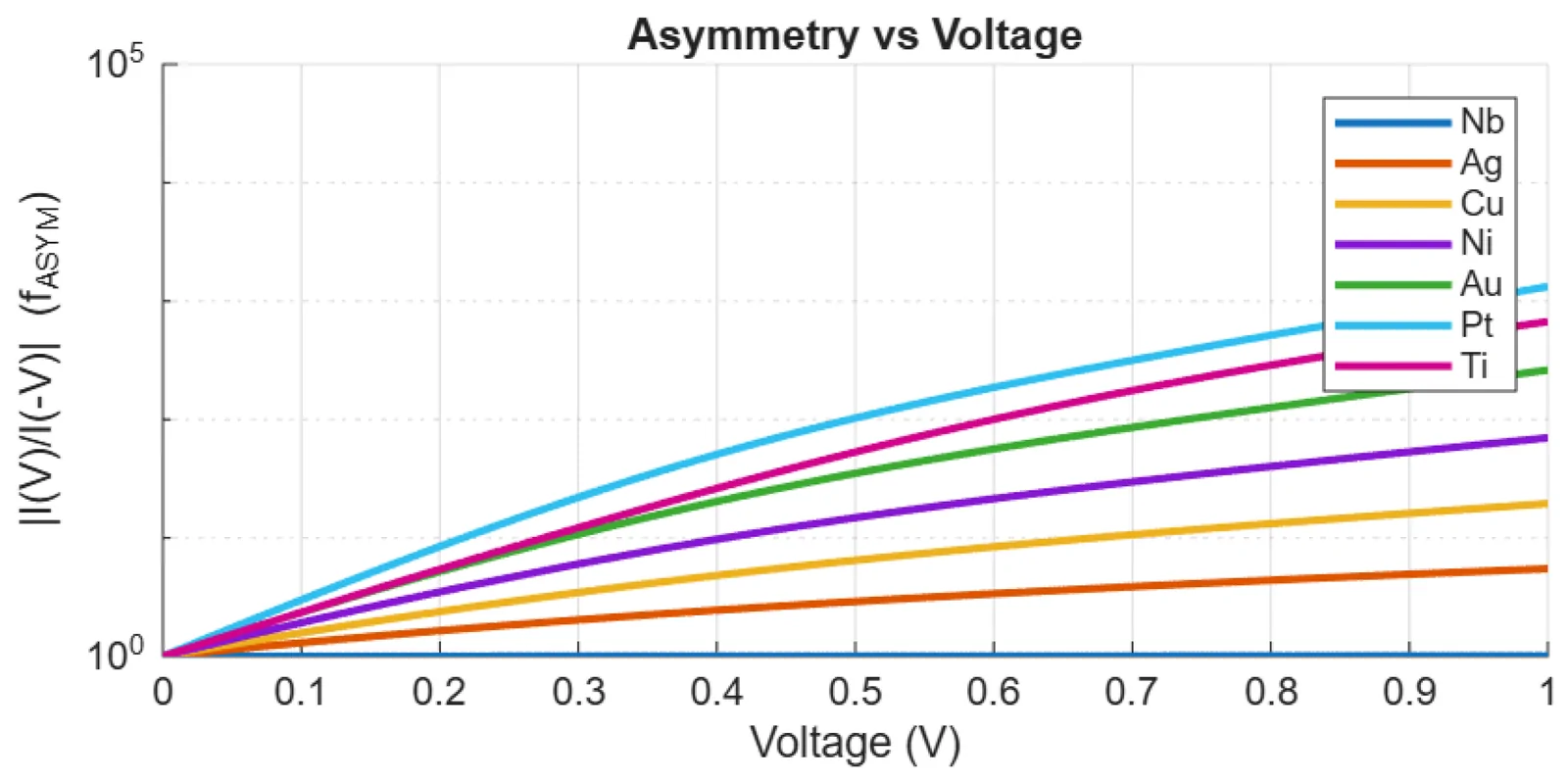
Asymmetry as a function of the electrode work-function difference ($\Delta \Psi \;=\;\Psi_{M1}-\Psi_{M2}$) for various material combinations.

Figure 10 illustrates the dependence of the asymmetry f_ASYM_ on the electrode work-function difference, ΔΨ, over the investigated voltage range of 0–1 V. As ΔΨ increases, the energy-band alignment at the two metal–insulator interfaces becomes increasingly asymmetric, resulting in a larger difference between the corresponding tunneling-barrier profiles. This asymmetry leads to different tunneling probabilities under opposite bias polarities, favoring electron transport in one direction while suppressing the reverse current. Consequently, f_ASYM_ increases with ΔΨ, demonstrating the important role of electrode work-function engineering in controlling the rectification behavior of the MIM junction.

Among the investigated electrode combinations, the Pt–Ti configuration exhibits the strongest asymmetric transport, with an asymmetry of approximately 2.5 × 10^4^. Nevertheless, a high f_ASYM_ alone does not fully characterize the performance of an MIM diode. It should therefore be considered together with other key FOMs, including zero-bias differential resistance, nonlinearity, responsivity, and junction capacitance. Their combined optimization is essential for achieving efficient electromagnetic-to-DC conversion [17].

**Figure 11:**
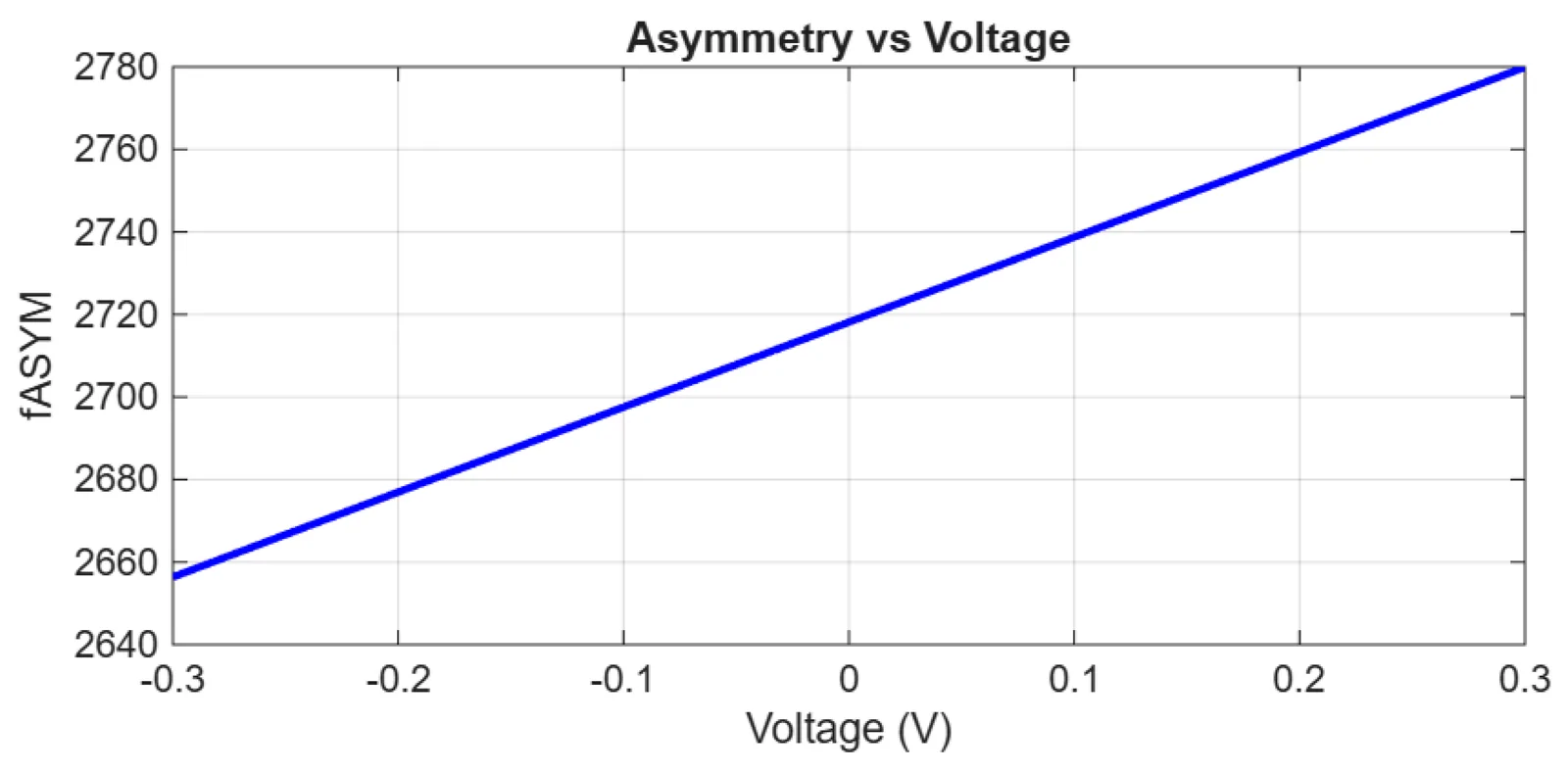
Asymmetry observed at 300 K and plotted as a function of the voltage in the range from −0.3 V to +0.3 V.

Figure 11 shows an asymmetry of 2720, confirming the intrinsic rectification capability of the proposed diode.

### 4.6. Non-Linearity $\left(f_{\mathrm{NL}}\right)$

**Figure 12:**
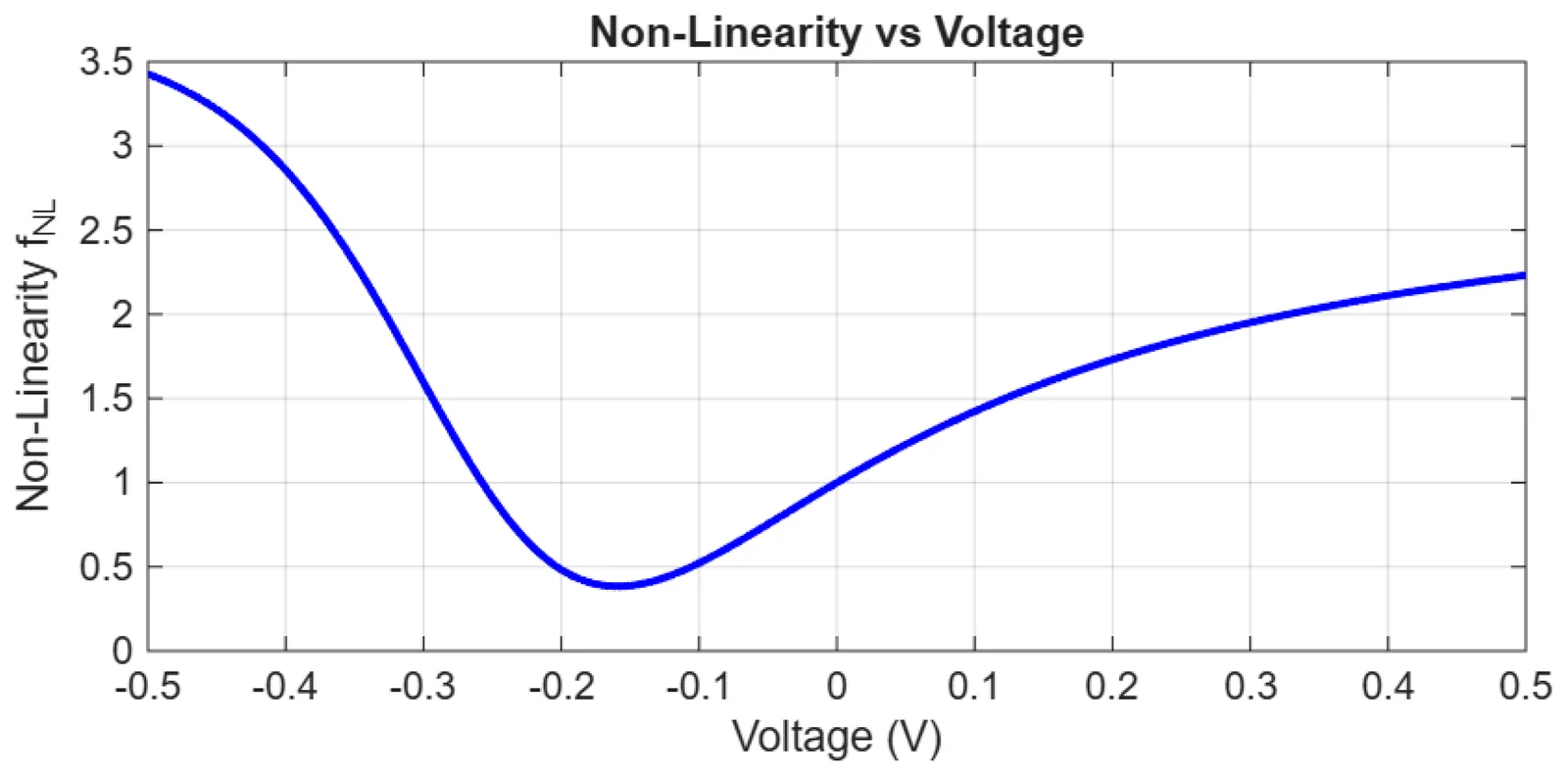
Non-linearity vs. Voltage (V) of the proposed Pt/HfSiON/Ti MIM Diode.

Figure 12 illustrates the nonlinearity (f_NL_) as a function of the applied voltage.

In the low-bias regime (∣V∣ < 0.1 V), the tunneling current exhibits an approximately linear dependence on the applied voltage, indicating that the electron transmission probability varies only weakly with bias. As the applied voltage increases, the I–V characteristic progressively departs from linearity due to the stronger bias-induced modification of the tunneling-barrier profile [17]. Accordingly, the nonlinearity f_NL_ should be regarded as a bias-dependent figure of merit rather than as a single parameter describing the entire I–V characteristic.

Near zero bias, f_NL_ approaches unity because the current is locally dominated by its first-order voltage dependence. This behavior reflects the quasi-linear response of the tunneling junction around the zero-bias operating point and should not be interpreted as an indication of poor rectification. At higher bias, the increasing contribution of higher-order terms in the tunneling current produces stronger I–V nonlinearity and, consequently, larger f_NL_ values. Thus, the near-unity f_NL_ observed at zero bias represents a local property of the tunneling response, whereas the increase in f_NL_ with bias reflects the progressive onset of nonlinear charge transport.

For the proposed Pt/HfSiON/Ti MIM diode, the rectification performance should therefore be assessed through the combined analysis of fNL, current asymmetry, and responsivity, rather than from the zero-bias value of f_NL_ alone.

### 4.7. Responsivity *(S)*

The responsivity formulation reported in Table 3 provides a direct measure of the diode’s ability to convert a small AC excitation into a rectified DC response. In particular, the zero-bias responsivity, S(0), is especially relevant for self-biased rectennas, where the incident electromagnetic radiation induces an AC voltage across the MIM junction without the need for an external DC bias. A high S(0) reflects a pronounced curvature of the I–V characteristic around the zero-bias operating point, indicating that the diode can effectively generate a DC response from weak electromagnetic excitations.

**Figure 13:**
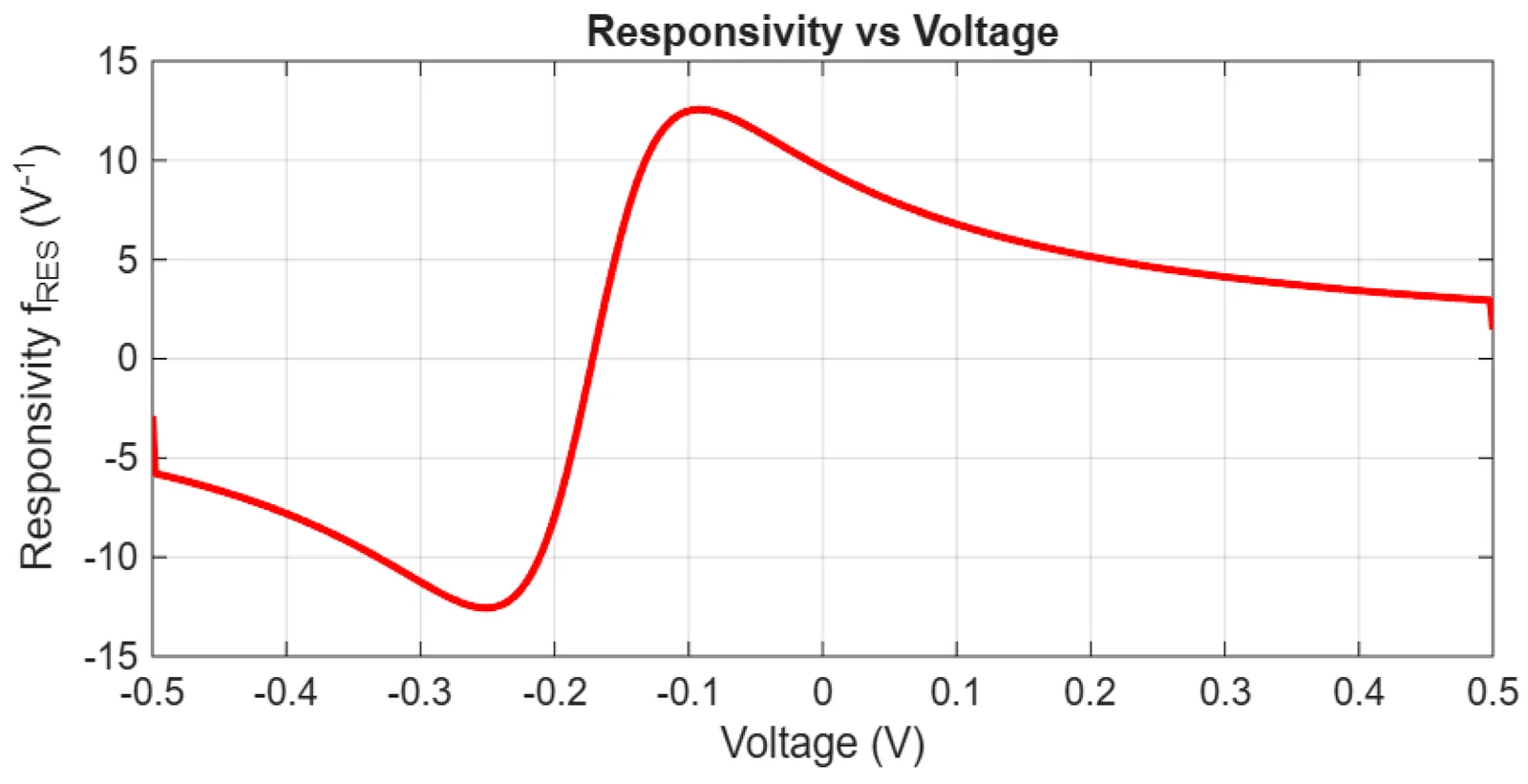
Responsivity versus voltage of the proposed Pt/HfSiON/Ti MIM Diode.

As shown in Figure 13, the responsivity reaches a maximum value of approximately 10 V^−1^ at zero applied DC bias. The responsivity remains close to 7^−1^ V at an applied bias of 0.1 V, demonstrating that the diode maintains a substantial nonlinear response over the low-bias regime.

The combination of high zero-bias responsivity and sustained sensitivity at small applied voltages highlights the potential of the proposed MIM junction for efficient self-biased rectification and low-power LWIR/THz energy harvesting. Nevertheless, the responsivity should be considered together with the zero-bias differential resistance, asymmetry, junction capacitance, and antenna–diode impedance matching, which collectively determine the practical IR-to-DC conversion performance of the rectenna [17,25,26,27,28,29,30,31].

### 4.8. Experimental Validation of Quantum-Tunneling Models

Experimentally measured current–voltage (I–V) characteristics provide a critical benchmark for assessing the physical validity and predictive capability of quantum-mechanical tunneling models for MIM and related nanoscale diode structures. A meaningful comparison between experimental and calculated I–V characteristics should extend beyond the absolute current magnitude and include the voltage dependence of the current, dynamic resistance, nonlinear curvature, polarity-dependent asymmetry, and the evolution of these quantities over the relevant bias range.

For a physically consistent tunneling model, the calculated transport characteristics should reproduce the experimentally observed current levels and bias dependence using parameters that are either independently measured or physically justified, including the effective junction area, dielectric thickness, barrier height, electron effective mass, and electrode work functions. Such a comparison provides a direct assessment of whether the assumed barrier profile and tunneling formalism adequately describe the actual junction [32].

Conversely, systematic discrepancies between experimental and theoretical I–V curves can provide important information about the limitations of the idealized transport model and may indicate the contribution of physical effects that are not explicitly included. These may include interface states, localized defects, spatial variations in barrier thickness or height, image-force-induced barrier lowering, electrode roughness, thermally assisted transport, trap-assisted tunneling, or resonant transport through localized states. Therefore, deviations should not necessarily be interpreted simply as model inaccuracies; they can also provide insight into non-idealities and additional transport mechanisms present in the experimental device.

This approach is illustrated by the experimental study of Belkadi et al., who compared measured and simulated I–V characteristics of Ni/NiO/Al_2_O_3_/Cr/Au metal–double-insulator–metal diodes using a quantum-transport model based on the solution of the Schrödinger equation and the calculation of the corresponding tunneling transmission probability. Experimentally determined junction dimensions and dielectric thicknesses were incorporated into the simulations, while the influence of key transport parameters, including barrier heights, effective mass, and effective electrical barrier thickness, was investigated to reproduce the measured electrical response.

The comparison further demonstrated that deviations between the idealized calculations and experimental data can be associated with interfacial effects and thickness-dependent variations in the dielectric layers that are not fully captured by a simplified barrier model. Accordingly, direct comparison with experimental I–V measurements represents an essential step in validating quantum-tunneling models for MIM junctions. Such validation not only establishes whether the model can quantitatively reproduce the experimentally accessible transport characteristics, but also helps identify the physical parameters and non-ideal transport mechanisms that must be considered to achieve a realistic description of nanoscale tunneling devices [32].

## 5. Comparative Analysis and Physical Interpretation of MIM Diode Performance

Table 4 presents a comparative assessment of the proposed MIM diode and previously reported devices operating at 28.3 THz, focusing on their device architectures, insulating materials, and key FOM.

## 6. Conclusions

A comprehensive theoretical and numerical investigation of a single-insulator Pt/HfSiON/Ti MIM diode for LWIR rectification at 10.6 μm (28.3 THz) has been presented. Building on the established MIM rectifier concept and recent advances in asymmetric tunneling junctions, the proposed vertical architecture combines dissimilar metal electrodes with an ultrathin HfSiON dielectric to engineer the interfacial energy-band alignment and enhance the asymmetry of quantum-mechanical tunneling. The resulting barrier asymmetry provides a means of controlling the bias-dependent electron transmission and, consequently, the nonlinear and rectifying response of the junction. Using the full Simmons tunneling model, the proposed Pt/HfSiON/Ti MIM diode exhibits a current density of 1.78 × 10^2^ A/cm^2^ at zero bias, increasing to 6.32 × 10^5^ A/cm^2^ at ±0.5 V. The calculated zero-bias current of 1.30 × 10^−9^ A confirms the presence of direct quantum-mechanical tunneling through the ultrathin HfSiON barrier. Under applied bias, the junction exhibits pronounced polarity-dependent transport, with forward and reverse currents of 4.61 × 10^−6^ A and 1.84 × 10^−10^ A, respectively, together with a zero-bias differential resistance of 3.85 × 10^5^ Ω. At 300 K, the diode combines several characteristics relevant to passive LWIR/THz rectification, including an asymmetry exceeding 2.5 × 10^4^, an asymmetry of approximately 2720 at ±0.3 V, and a peak zero-bias responsivity of approximately 10 V^−1^, while maintaining a responsivity of about 7 V^−1^ at 0.1 V. The high zero-bias responsivity, together with the pronounced current asymmetry, indicates strong potential for self-biased rectification, in which incident LWIR radiation can be converted into a net DC response without an externally applied bias. The near-unity nonlinearity observed around zero bias should be interpreted as a local characteristic of the tunneling response rather than as an indicator of the overall rectification capability, which is governed by the combined behavior of nonlinearity, current asymmetry, responsivity, and differential resistance. The calculated performance can be attributed primarily to the asymmetric energy-barrier profile established by the dissimilar Pt and Ti electrodes. The resulting difference in electrode work functions produces unequal interfacial barrier heights and, consequently, strongly different electron transmission probabilities under opposite bias polarities. At the same time, the ultrathin HfSiON layer provides a sufficiently narrow tunneling barrier to sustain substantial electron transmission, thereby enabling a favorable balance between tunneling current and rectification. These results highlight the importance of electrode work-function engineering, barrier-height asymmetry, and dielectric-layer properties in tailoring the transport characteristics of MIM junctions. In this context, HfSiON represents a promising dielectric platform for high-frequency MIM rectifiers and LWIR/THz rectenna architectures.

Overall, the numerical results identify the Pt/HfSiON/Ti junction as a promising candidate for passive, self-biased LWIR energy harvesting. Nevertheless, the practical performance of an integrated rectenna cannot be determined from diode characteristics alone and will depend on the simultaneous optimization of diode nonlinearity, asymmetry, zero-bias differential resistance, junction capacitance, and antenna–diode impedance matching. Future work will therefore focus on experimental fabrication and electrical characterization of the proposed junction, followed by its integration with nanoscale LWIR antenna architectures. Experimental assessment of antenna–diode coupling, impedance matching, and overall IR-to-DC conversion efficiency will be essential to establish the practical viability of HfSiON-based MIM rectifiers for compact, self-powered infrared energy-harvesting systems.

## Figures and Tables

**Table 1:** Electrical performance analysis of the MIM diode and corresponding FOM [17].

FOM	Definition	Key Notes/Trade-Offs
Zero-Bias Dynamic Resistance ($R_{0}$) Ω $R_{0}\;=\;{\left(\frac{\mathrm{dI}}{\mathrm{dV}}\right)}_{V\;=\;0}^{-1}$	Small-signal resistance at zero bias, characterizing the differential V–I response near the operating point.	Determines the antenna–rectifier impedance matching by optimizing the barrier height and thickness.
Asymmetry ($f_{\mathrm{ASYM}}$) $f_{\mathrm{ASYM}}\left(V\right)\;=\;\frac{I_{\mathrm{ON}}}{I_{\mathrm{OFF}}}\;=\;\left|\frac{I_{F}\left(V\right)}{I_{R}\left(V\right)}\right|$	Quantifies the imbalance between the forward IF and reverse currents IR, reflecting the diode’s rectification capability.	Values greater than unity indicate enhanced rectification, which can be tuned by controlling the work function difference between the metal electrodes.
Nonlinearity ($f_{\mathrm{NL}}$) $f_{\mathrm{NL}}\left(V\right)\;=\;\frac{\frac{\mathrm{dI}}{\mathrm{dV}}\left(V\right)}{\frac{I(V)}{V}}$	Quantifies the degree of nonlinearity of the diode’s I–V characteristic, reflecting its deviation from linear conduction.	Values greater than 3 indicate strong rectification, governed by the barrier geometry, thickness, and degree of asymmetry.
Responsivity ($f_{\mathrm{RES}}$) V^−1^ $f_{\mathrm{RES}}\left(V\right)\;=\;\frac{\frac{d^{2}I}{d^{2}V}\left(V\right)}{\frac{\mathrm{dI}}{\mathrm{dV}}\left(V\right)}$	Quantifies the efficiency of converting incident electromagnetic energy into electrical energy.	Values greater than 7 indicate efficient zero-bias rectification, enhanced by barrier asymmetry and optimized barrier thickness.
TOV	Minimum bias voltage required to achieve substantial quantum tunneling through the insulating barrier.	Low values are desirable for IR rectennas and can be achieved using ultrathin, low-bandgap barriers with optimized barrier heights.

**Table 2:** Impact of Design Parameters on Cutoff Frequency and Junction Area.

fc	Ra	Cd	$\varepsilon_{0}$	$\varepsilon_{r}=k$	A	d
28.3 THz	300 Ω	1.9 × 10^−17^ F	8.854 × 10^−12^ F/m	10	21 nm × 21 nm = (430 nm^2^)	2 nm

**Table 3:** Design parameters of the Pt/HfSiON/Ti MIM diode.

Parameter	Value
Work function of Pt $\left(\Psi_{1}\right)$	5.6 eV
Work function of Ti $\left(\Psi_{2}\right)$	4.33 eV
Electron affinity of HfSiON $\left(\chi \right)$	4.0 eV
Bandgap of HfSiON	5.5 eV
Left barrier height $\left(\Phi_{L}\;=\;\Psi_{1}-\chi \right)$	1.65 eV
Right barrier height $\left(\Phi_{R}\;=\;\Psi_{2}\;-\;\chi \right)$	0.33 eV
Work-function difference $\left(\Delta \Psi \right)$	1.33 eV
Breakdown voltage V_BD_$\;\approx \;2\left(\Psi_{1}\;-\;\Psi_{2}\right)$	2.64 V
Insulating layer thickness d	2 nm

**Table 4:** Comparison of the proposed MIM diode with previously reported devices at 28.3 THz.

Material	Cut-Off Frequency	Thickness	J_ON_	f_ASYM_	f_NL_	f_RES_ (V^−1^)	Zero Bias f_RES_ (V^−1^)
Cu (100 nm)-CuO-Au (100 nm) (0.0045 μm^2^) [32]	28.3 THz	CuO (0.7 nm) Au/Cu (100 nm)	-	-	-	6	4
Ti-TiO_2_-Al (21,287 µm^2^) [33]	Up to 150 THz	TiO_2_ (9 nm)	10^−1^ A/cm^2^	-	6.5	18	-
Ti-TiO_2_-Pt (21,287 µm^2^) [34]	Up to 150 THz	TiO_2_ (9 nm)	10^−0^ A/cm^2^	-	15	15	-
Nb/Nb_2_O_5_/Pt [35]	Up to 150 THz	Nb_2_O_5_ (15 nm)	-	1500	4	20	-
Nb/Nb_2_O_5_/Cu [35]	Up to 150 THz	Nb_2_O_5_ (15 nm)	-	1500	8	20	-
Nb/Nb_2_O_5_/Ag [35]	Up to 150 THz	Nb_2_O_5_ (15 nm)	-	1500	8	20	-
Nb/Nb_2_O_5_/Au [35]	Up to 150 THz	Nb_2_O_5_ (15 nm)	-	1500	8	20	-
Au/Al_2_O_3_/Pt [35]	Up to 28.3 THz	Al_2_O_3_ (1.4 nm) Au/Pt (100 nm)	-	-	6	-	10
Ni-NiO-Ag (3.1 × 10^−4^ µm^2^) [28]	Up to 343 THz	NiO (6 nm)	-	5	3	8.5	5.8
Pt-SiCl_3_-(CH_2_)_17_-CH_3_ -Ti (100 μm^2^) [36]	Up to 150 THz	SiCl_3_-(CH_2_)_17_-CH_3_ (2.23 nm)	-	117.8	6.8	20.8	8.0
Nb/TiO_2_/Pt [33]	Up to 30 THz	TiO_2_ (13 nm)	-	80	3.5	-	-
Nb/Nb_2_O_5_/Ni [33]	Up to 150 THz	Nb_2_O_5_ (15 nm) Nb/Ni (90–100 nm)	1 × 10^−10^ A/cm^2^	396.5	7.1	8.5	-
Nb/Nb_2_O_5_ (15 nm)/Au [33]	Up to 150 THz	Nb_2_O_5_ (15 nm) Nb/Au (90–100 nm)	-	1430.8	8.0	7.0	-
SrTiO_3_ (STO)/Al_2_O_3/_SrTiO_3_ (STO) [37,38]	Up to RF	-	5 × 10^−9^ A/cm^2^	-	-	-	-
Cu-CuO-Cu (2 × 2 μm^2^) [39]	Up to 150 THz	CuO (2 nm) Cu (100 nm)	-	-	-	4.497	-
Pt/Al_2_O_3_/Al [40]	Up to 150 THz	Al_2_O_3_ (6 nm) Pt/Al (100 nm)	-	110 for AP-CVD 30 for PEALD	6 for AP-CVD 30 for PEALD	9 for AP-CVD 22 for PEALD	-
Al-Al_2_O_3_-Au [41]	Up to 60 THz	Al/Au (65 nm)	4.0 μA/cm^2^	-	-	14.46	-
Al-Al_2_O_3_-Cr [42,43]	Up to 28.3 THz	Al_2_O_3_ (3 nm) Al/Cr (100 nm)	2 × 10^−4^ A/cm^2^	-	3.1	-	-
This work Pt/HfSiON/Ti	Up to 28.3 THz	Pt(100 nm)/HfSiON (2nm)/Ti(100 nm)	1.78 × 10^2^ A/cm^2^	2.5 × 10^4^	1	7	10

## Data Availability

The original contributions presented in this study are included in the article. Further inquiries can be directed to the corresponding author.

## Abbreviations

The following abbreviations are used in this manuscript:

LWIR	Long-Wave Infrared
THz	Terahertz
MIM	Metal-Insulator-Metal
FOM	Figure of Merit
IR	Infrared
DC	Direct Current
AC	Alternating Current
R_0_	Zero Bias Resistance
Ψ	Work Function
χ	Electron Affinity
Φ	Barrier Height
ε_r_	Relative Dielectric Constant
ε_0_	Permittivity of Free Space
V	Bias Voltage
d	Dielectric Thickness
A	Device Area
fc	Cutoff Frequency
R_D_	Dynamic Resistance
C_D_	Junction Capacitance
ηc	Antenna-Diode Coupling Efficiency
ΔΨ	Work-Function Difference
f_ASYM_	Asymmetry
f_N_L	Nonlinearity Factor
f_RES_	Responsivity
TOV	Turn-On Voltage
V_0_	Zero Bias
I–V	Current-Voltage
J–V	Current Density-Voltage
